# Transient Behavior in Variable Geometry Industrial Gas Turbines: A Comprehensive Overview of Pertinent Modeling Techniques

**DOI:** 10.3390/e23020250

**Published:** 2021-02-22

**Authors:** Muhammad Baqir Hashmi, Tamiru Alemu Lemma, Shazaib Ahsan, Saidur Rahman

**Affiliations:** 1 Department of Mechanical Engineering, Universiti Teknologi PETRONAS, Seri Iskandar, Perak Darul Ridzuan 32610, Malaysia; baqirhashmi.123@gmail.com (M.B.H.); shazaibahsan@hotmail.com (S.A.); 2 Research Center for Nano-Materials and Energy Technology (RCNMET), School of Science and Technology, Sunway University, Bandar Sunway, Petaling Jaya 47500, Malaysia; saidur@sunway.edu.my; 3 Department of Mechanical and Manufacturing Engineering, Faculty of Engineering, Universiti Putra Malaysia, Serdang 43400, Malaysia

**Keywords:** fault detection and diagnostics, industrial gas turbine, transient model, variable geometry, variable inlet guide vanes

## Abstract

Generally, industrial gas turbines (IGT) face transient behavior during start-up, load change, shutdown and variations in ambient conditions. These transient conditions shift engine thermal equilibrium from one steady state to another steady state. In turn, various aero-thermal and mechanical stresses are developed that are adverse for engine’s reliability, availability, and overall health. The transient behavior needs to be accurately predicted since it is highly related to low cycle fatigue and early failures, especially in the hot regions of the gas turbine. In the present paper, several critical aspects related to transient behavior and its modeling are reviewed and studied from the point of view of identifying potential research gaps within the context of fault detection and diagnostics (FDD) under dynamic conditions. Among the considered topics are, (i) general transient regimes and pertinent model formulation techniques, (ii) control mechanism for part-load operation, (iii) developing a database of variable geometry inlet guide vanes (VIGVs) and variable bleed valves (VBVs) schedules along with selection framework, and (iv) data compilation of shaft’s polar moment of inertia for different types of engine’s configurations. This comprehensive literature document, considering all the aspects of transient behavior and its associated modeling techniques will serve as an anchor point for the future researchers, gas turbine operators and design engineers for effective prognostics, FDD and predictive condition monitoring for variable geometry IGT.

## 1. Introduction

Nowadays, gas turbines (GTs) are being commonly used in combined cycle power plants (CCPPs) for power generation and mechanical drive applications in the oil and gas industries. The compactness, light weightiness and acceptability for a variety of fuels make GTs suitable for offshore utilities [1,2,3]. Apart from that, the lower manufacturing cost, design flexibility, lower start-up time, lower maintenance and environment friendly features endorse the gas turbine as a greater priority over the steam turbine. For power generation purposes GTs are also used in CCPP mode. In this way, GTs provide an enhanced efficiency of about ~55%, while simple steam turbine is limited to ~35% [4]. Among the fossil fuel-based power plants, gas turbine stands first in terms of high efficiency, greater size ranges of power output and less operation and maintenance cost. Generally, gas turbines are classified as aviation, stationary, and marine gas turbines. Aviation gas turbines are commonly used as aircraft propulsion systems to provide thrust to the airplane during flight. To date, several modifications have been made on the turbofan engines to use them for industrial applications [5,6].

Gas turbines have certain design conditions, however, they are being operated off design due to power fluctuations, which results in performance degradation [7,8]. Apart from this, some critical factors such as startup, load change, shutdown, variations in ambient conditions, equipment failure and other abnormal behavior exist, which can trigger transient behavior by shifting the engine’s equilibrium from one steady state to another steady state [9]. This can lead to certain thermal, aerodynamic and mechanical stresses in the gas turbine, which are responsible for reduced availability, poor reliability and drastically increased maintenance cost [10]. Therefore, these critical factors should be accurately modeled to ensure enhanced availability, higher reliability, safe operation and reliable control. In this regard, a transient simulation model is indispensable for stable operation, high fidelity controller design, accurate real time fault detection and diagnostics (FDD). 

Increased demand for electricity generation and global economic vulnerability, urges an operational flexibility for new generation gas turbines, which can provide a fast response against any variation in power demand. Engine manufacturing firms are emphasizing on fast operations (start-up, load ramp up and shutdown) to achieve better fuel economy and reduced emissions as compared with conventional turbines. Nowadays, the pursuit of sustainability, has paved the way for gas turbines to be integrated with renewable energy sources as a hybrid system. However, some limitations exist for this system that can cause several aero-thermal and mechanical stresses leading to equipment failure. Therefore, the current dynamic and flexible operational profile of gas turbine needs transit model development.

**Figure 1:**
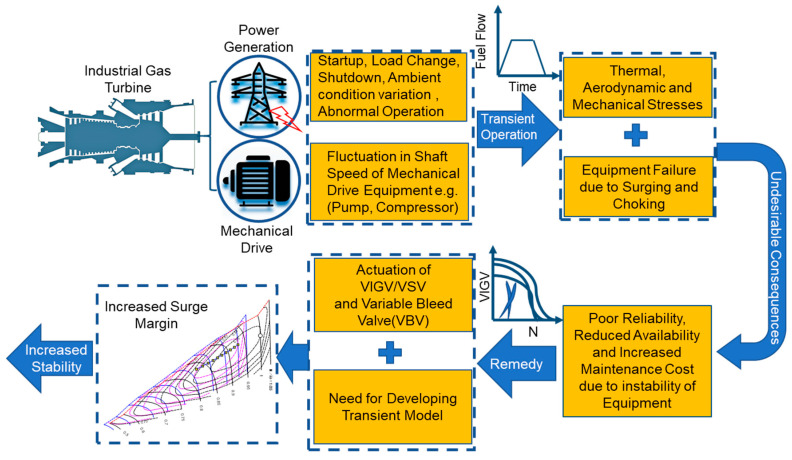
Role of variable geometry inlet guide vanes (VIGVs) in transient behavior.

The transient factors that are leading to instability of the engine can be rectified by incorporating variable geometry features in the gas turbines, i.e., variable inlet guide vanes (VIGVs), variable stator vanes (VSV), variable bleed valve (VBV), and variable area nozzle (VAN). In this case, the instability of compressor in form of surging and choking can be reduced using the modulation of VIGVs [11,12,13,14,15]. A group of researchers have well reviewed the transient models revealing their scope in variety of applications such as system performance analysis, fault identification, effective controller design, condition monitoring, diagnostics and prognostics purposes [16]. The pursuit to attain a stable operation during transient behavior incorporating variable geometry feature has been idealized via a pictorial representation shown in Figure 1. 

### 1.1. Literature Survey of Transient Modeling Domains 

The established transient models find scope in variety of applications such as system performance analysis, fault identification, effective controller design, condition monitoring, diagnostics and prognostics purposes as reviewed by [16]. The reason for developing the transient model lies in the fact that, during transient operation, engine’s life deteriorates more drastically than that of a steady sate base load engine. A variety of pertinent literature is available for transient modeling of various kinds and configurations of engines such as industrial gas turbines, aero gas turbines and marine engines. Transient modeling domains are further discussed in the following passages.

An in depth literature review manifests that, the majority of studies related to the transient model have been carried out in order to develop control system strategies, since control is the most indispensable entity during transient operation for insurance of stable engine operation. Earlier researchers have established control system simulators for investigation of dynamic behavior of aero engines. They were based on simple block diagram and generalized type programs [17]. Although, these simulators were very simple and easy to develop but entailed limitations in dynamic studies considering few dynamic variables. Moreover, generalized type simulators proved to be time consuming in solving the Jacobian matrix. Another implemented control system technique was closed loop proportional-integral-derivative (PID) control scheme to capture the entire transient operation in mechanical drive GT power station [18]. The transient model in this study was complex and tedious to develop, since it covered the various auxiliary components of the power station. However, it emerged as a holistic model and benefitted in monitoring the surging, startup and slow transient operation occurring inside the centrifugal compressor section. In the meantime, Badmus et al. [19,20] came up with idea of developing independent turbomachinery transient models to observe the instabilities arising in the turbomachinery components [21]. However, this model was limited to merely one-dimensional unsteady flow of the compressor and was lacking implementation for the two-dimensional model that is needed for designing a surge control and stall avoidance schemes. Moreover, the model was not fully validated with the test rig data. Lichtsinder and Levy [22] proposed an improved and advance digital modeling method named as novel generalized describing function (NGDF). This quasi-linear control model could cater the large transient variations in operational envelope with a less computational time. 

Recently, Tsoutsanis and Meskin [23] have developed a dynamic model for hybrid gas turbine and wind turbine system in order to design a controller and optimize the operation in hybrid mode. Similarly, Park [24] has also developed a hybrid dynamic model of a distributed energy system having small gas turbine, diesel engine, fuel cell, solar source and synchronous machine. Several other researchers have established dynamic models for design of controller and effective control strategy for actuation of VIGVs and fuel valves [25,26,27,28,29,30,31]. Similarly, Kong and Kim [32] have focused on performance optimization and controller design of a turbojet engine. Bettochi et al. [33] stated a transient modeling study for control system diagnosis of a single shaft industrial gas turbine. Mehrpanahi et al. [34] utilized the developed dynamic model to estimate the revolution per minute (RPMs) of shaft during start up and load change phases using conditions monitoring data. The current pursuit of higher reliability, increased availability and reduced maintainability have motivated various researchers to incorporate transient models for fault diagnostics [35,36], engine performance monitoring [37], and performance prediction associated with diagnostics [38,39]. Moreover, some other authors have conducted the transient modeling study in order to achieve optimization in terms of several perspective such as part load performance optimization [40] and optimization of VIGV and bleed extraction and stall domain prediction [41]. 

In addition to the above-mentioned purposes, effects of fuel control on transient behavior and combustion chamber’s transients have also been studied. For instance, Ma et al. [42] has developed a transient model for the development of fuel control strategy for the starter of gas turbine. Likewise, Wang et al. [43] have studied the effect of incorporation of fuel control system along with the generic control system, on the time delay during transient behavior. Additionally, Singh et al. [44] has investigated the effect of variation in the fuel’s lower heating value on the transient behavior. 

Apart from this, Kim et al. [45] has utilized their developed transient model to see the effect of time lag during fuel flow and VIGV control. Metzger [46] has developed a dynamic simulation in order to test a dry low NOx prototype turbojet engine before commercialization. The focus of this research remained toward verification of the fuel control system. Rosfjord and Cohen [47] have suggested and utilized a new test facility to evaluate the transient behaviors occurring in the combustor. The study proved to be helpful for estimation of air and fuel flow time variation rates along with air temperature. The others motives that incited researchers for transient simulations are performance prediction [32,48,49,50,51,52] and compressor and nozzle performance maps evaluation [53]. The modeling and simulation of an engine involve various other factors that influence the transient behavior through one or other way. For instance, Shi et al. [54] has done transient performance simulation to observe the effect of compressibility on the transient behavior while Novikov [55] studied the effects of inlet pressure distortion and component deterioration on the transient operation. 

Hence, an in-depth scrutiny of the literature manifests that transient studies of aero engines has remained the cornerstone for the majority of the researchers; whereas transient models for variable geometry industrial gas turbines are rarely available. However, IGTs associated with CCPPs can be observed in some instances. It can be implicated that transient modeling in the context of variable geometry IGT remained slightly under focused. However, a list of few studies involving transient behavior of variable geometry gas turbines is stated in Table 1. Among the variable geometry features adopted in these transient modeling studies, majority of the researchers emphasized on the incorporation of VIGV/VSV while few authors have considered variable bleed valves (VBVs) and nozzle guide vanes (NGVs). The apparent reason lies in the fact that it is very hard to find VBV schedules in the public domain. Although, a few researchers have taken care of VBV, but the schedules have been overlooked throughout the course of history. 

### 1.2. Research Gaps 

After extensive investigations on the already published literature, it became evident that:

- To the author’s best knowledge, there is a lack of organized literature review so far that may cover all the possible techniques and methods for the development of transient models of industrial gas turbine regarding fault detection and diagnostics (FDD).
- The pertinent literature for variable geometry features (i.e., VIGVs, VSVs, variable bleed and VAN) that play significant role in engine’s reliability preventing engine from surging during transient behavior, remained shallow.
- There is no such existing document that provides accurate data for shaft’s polar moment of inertia required for accurate transient model development
- To date, there is no authentic document that aids in selection of proper VIGV and bleed schedules for a particular IGT engine based on its inherent configuration, i.e., single shaft, twin shaft, and triple shaft

These research gaps paved the way to develop a holistic documentation that will help the future researchers in developing transient model for effective health monitoring of the industrial gas turbines. As such, in the present work, an extensive literature review has been conducted to build a foundation for developing transient model for variable geometry industrial gas turbine. Detailed literature study has manifested that, although variety of pertinent literature exists regarding the dynamic simulation of gas turbines, variable geometry gas turbines transients remained slightly under focused. As the complete modeling of the industrial gas turbine is an embodiment of the modeling of its constituent components—that is why this review is covering all the methodologies involved in the modeling of constituent components of the industrial gas turbine. Moreover, this review formulates a classification for a variety of VIGV and bleed schedules explored from the literature. Additionally, data relevant to shaft polar moment of inertia for variety of configuration of engines have been collected in this review paper. Hence this review will serve as a supporting document for selection of a best VIGV schedule, bleed schedule and authentic shaft polar moment of inertia for developing transient models of any configuration engine.

## 2. Classifications of Transient Regimes in IGT

**Figure 2:**
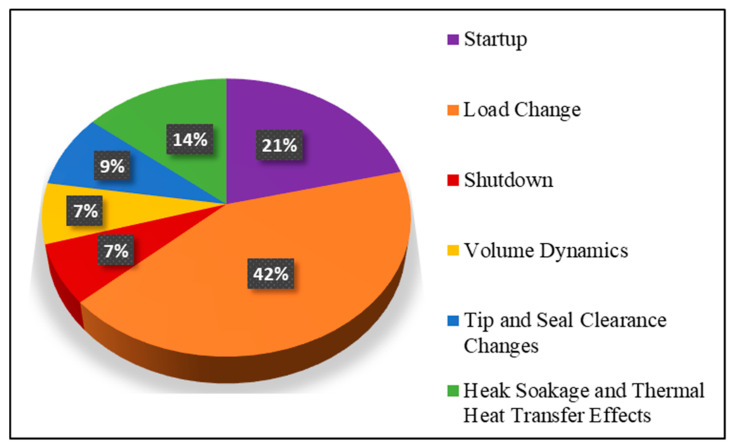
Classification of transient behaviors based on literature.

**Figure 3:**
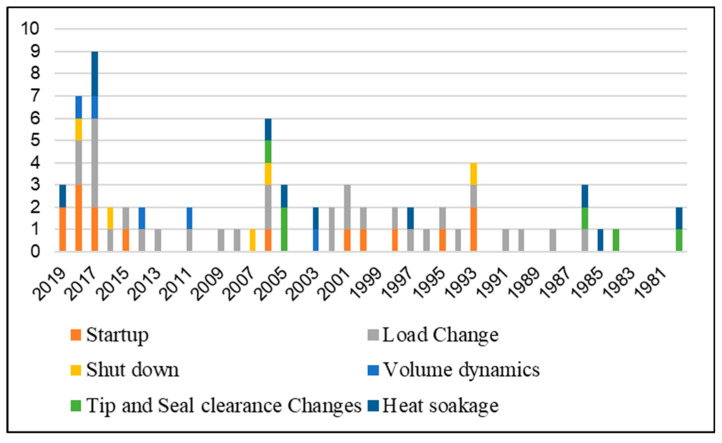
Historical trend of various transient regimes in the literature.

Transient behavior in an industrial gas turbine is usually occurred during its startup, load change, shutdown and ambient conditions variation. However, some other phenomena that are not very common such as over speeding due to shaft failures, emergency shutdown and sudden load drop, can also trigger transient behavior. Apart from this, there are some secondary effects that may also lead to transient operation such as volume packing, tip and seal clearance changes, combustion delay and control system lag. The detailed transient regimes are discussed in the following subsections. A literature based statistical segregation of different phenomena causing transient behavior are illustrated in Figure 2 and Figure 3. The illustration in Figure 3 represents the focus and emphasis of the various transient modeling studies. It has been analyzed that load change transient phenomenon has been remained the abundantly focused area among most researchers. However, volume dynamics followed by tip and seal clearance changes and heat soakage and thermal heat transfer effects are the least addressed subjects in terms of transient modeling. On the other hand, the bar chart in Figure 3, portrays a historical trend of several transient modeling phenomenon with a segregated amount of the frequency of the transient studied happened in any particular year. Further details are stated in the following sections.

### 2.1. Startup 

**Figure 4:**
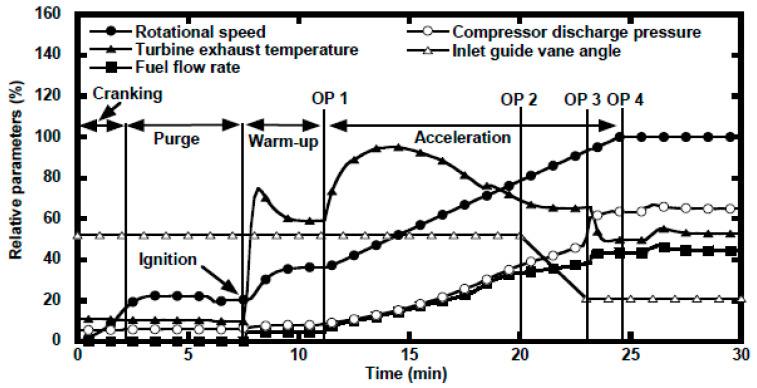
Different phases involved in startup operation [82].

The transient phenomenon at startup is very critical in terms of instability occurred due to surging, rotating stall and hot start. Gas turbine’s startup begins by cranking the engine shaft with any starter prime mover. The starter prime mover keeps on supporting the engine, generally up to a speed of around 40–85% of the rated speed, until the turbine’s efficiency is achieved high enough to become independent of the starter [79]. Different phases of startup operations are shown in Figure 4. At lower speeds, the compressor pressure ratio is lower and hence the density (pressure) reduction can increase the axial velocities at rear stages. Eventually, due to increased axial velocities, the mass flow is reduced and thus choking occurs [80]. During startup, the surge and stall can cause an anomalous tripping or shutdown due to lower speed. If the starter support becomes disengaged at that instant, the engine will decelerate while increasing the temperature rigorously and hence Hot Start is occurred [12]. Apart from this, high pressure ratio multistage axial flow compressor are usually encountered with performance deterioration, i.e., decreased pressure ratio and efficiency at lower speeds [81]. In order to avoid from such kind of mishaps, a transient model is needed for effective study of the fuel flow and starter cutoff schedule.

The gas turbine’s startup operation has been simulated by various authors over the course of history. Chappel et al. [83] developed a very first transient model in order to simulate the startup operation from zero speed to maximum rated power, but still the sub idle regime has never been addressed by him. Recently, Mohammadian and Saidi [56] have developed a transient model to study the complete startup operation of industrial twin shaft gas turbine. In order to ensure higher robustness and fidelity regarding control during startup transient, this model has considered the simultaneous control of VIGV, variable bleed valve and fuel flow valve. Zeng et al. [84] has also developed a startup transient model for fault detection and diagnostics using cuckoo search optimization algorithm. Similarly, Mehrpanahi et al. [34] have tried to estimate the shaft revolution rate during startup operation by establishing a transient simulation model suing condition monitoring data. Ma et al. [42] have conducted a study on the gas turbine starter and associated fuel control system modeling. Likewise Ghaffari et al. [85] have also developed a transient model for startup simulation of a heavy duty gas turbine of MAPNA organization situated in Iran.

In general, simulation of early stages of startup, from stationary phase to purging, is considered quite difficult because, at early phase of the starter the compressor characteristics maps are rarely available, and it is also very hard to generate compressor maps at sub-idle range. Sometimes map extrapolation techniques are adopted to estimate the characteristics of compressor and turbine at very low speed using already available component maps with low speed data [86]. However, this technique is not reliable and might cause problem in the convergence during iterations. Apart from this, there is no such simulation program commercialized so far which must possess the capability of simulating the startup at early stage of startup, i.e., zero rpms to idle power conditions. However, in a very recent research, Kim and Kim [82] have developed a dynamic simulation program in order to simulate the startup operation of a heavy duty IGT from zero speed to idling condition. Moreover, this program has the potential advantage of prediction of optimal starter capacity that can be determined by purge analysis. During startup, starter capacity manages two things, i.e., (i) startup time, and (ii) fuel flow schedule. Hence, an engine comprising of higher starter capacity is very beneficial in terms of fuel saving and preventing the hot parts of the engine from damage due to excessive fuel flow. Additionally, some other researchers have developed transient models for idle and sub-idle regimes for civil aero engines, such as sheng et al. [87], who suggested a stage stacking method for extrapolation of low speed region in order to simulate a full rage startup transient model for a turboshaft engine. Similarly, Kim et al. [88] have used a thermodynamic model for a triple-shaft turbofan engine in order to simulate three different kind of startup regimes, i.e., wind milling, sub-idle and idle to maximum power ranges.

### 2.2. Load Change (Acceleration and Deceleration)

In general, gas turbines come across dynamic behavior during load change. Load change normally involves the rapid increase and decrease of input fuel flow that leads the engine to a transient state. During transient operation, the work produced by the rotor shaft may exceed or recede from the work that is used to balance compressor and turbine respective work outputs [89]. Mostly, a rapid acceleration is responsible for frequent overshooting of turbine inlet temperature (TIT) that can create various thermal stresses in the turbine blade and eventually lead to blade failure. In the long run, over all heath of the gas turbine becomes shorter due to frequent accelerations and decelerations. Hence, a study that might cover all the transient characteristics of a gas turbines is of paramount importance to ensure reliable operation.

As far as load change in industrial gas turbine is concerned, it is varied in two ways: (i) part load in CCPPs and (ii) speed fluctuation in mechanical drive equipment. In power generation, IGTs are generally associated with heat recovery steam generators (HRSGs) in combined cycle power plants (CCPPs). Usually, part load scenario is faced in combined cycle power plants, where the load is controlled by fuel flow mostly. However, combined cycle power plants require a designated exhaust gas temperature to keep the effectiveness of HRSG at optimum level [90]. Load changing phenomenon in power generation as well as variable speed behavior in mechanical drive applications shift engine into off design operation [91]. In turn, excessive mechanical and thermal stresses are developed along with performance degradation. 

Several studies have been conducted for analysis of load change behavior of the gas turbines. The purpose of some the studies is merely model synthesis, i.e., only model development at certain operational behavior, while rest of the studies cover model analysis, i.e., FDD, prognostics, condition monitoring, and control system design as mentioned in the introduction section. Recently, Silva et al. [60] have developed a transient model for a three-shaft counter rotating open rotor (CROR) marine engine. The intended purpose of this research was to observe the effect of VIGV angle modulation on the load change transient, i.e., load increase (acceleration) and load decrease (deceleration), and incorporation of VIGVs resulted in increased surge margin during load increase phase. Lyantsev et al. [92] have studied the acceleration process of a turbojet engine to implicate a new system identification technique for the fast countable automatic control system of dynamic behavior of the gas turbine. The novelty in the work was to determine an acceleration parameter using a numerical optimization method to simulate an accurate acceleration mode using experimental data. Yamane [93] has also conducted one such similar study for turbofan engine to build a high fidelity nonlinear dynamic model that can evaluate the acceleration from idle to max range while incorporating time lag between fuel flow and actual combustion time. Similarly, Ki et al. [89] have developed a transient model for a turboshaft unmanned air vehicle (UAV) to check the effect of rapid acceleration and deceleration on the overall performance of gas turbine. This study showed a substantial increase in rotor speed and burner temperature due to rapid fuel variation. 

### 2.3. Shutdown

**Figure 5:**
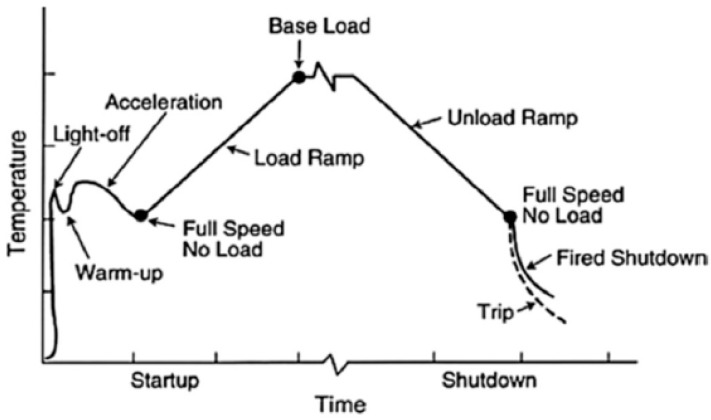
Temperature variation during startup and shutdown transients [94].

As far as shutdown of an industrial gas turbine is concerned, fuel flow schedule is always adopted for a sequential shutdown just like Startup trajectory. Fuel flow schedule is in the gradually decreasing trend with respect to time in order to reduce the generation level up to minimum power output. During shutdown several low cycle fatigue phenomenon, i.e., thermal fatigue stresses in the turbine casing, are developed that are caused by the time variant temperature gradients during turbine operating cycles [94]. These kind of temperature gradients can cause various vibrational problems that can lead to component failure. In order to evaluate these kind of stresses, dynamic models are the most effective method. Unfortunately, very little work has been put forth for developing transient models during shutdown operations. However, Chappel et al. [83] has developed a transient model both for startup and shutdown regimes. As the shutdown operation is typically more crucial in terms of thermo-mechanical cycle loads for IGTs, that is why Reddy et al. [95] has developed a shutdown model for IGTs in order to study the thermal effects involved in the transient operation. Although, development of transient model for evaluation of thermo-mechanical behavior during shutdown is very complex task because associated difficulties mentioned in the work, but this research work can be helpful for the future researchers. Likewise, Svensdotter et al. [96] have also developed a transient shutdown model to control the bearing soak back peak by developing a time based correlations between the temperature at shutdown and bearing soak back peak temperature. The effect of temperature fluctuation during startup and shutdown transients is shown in Figure 5. 

Apart from normal shutdowns, there is another phenomenon that can bring the gas turbine to tremendous dynamic instability that is emergency shutdown or sudden load rejection or load shedding. Load rejection can cause a catastrophic mechanical stress especially in multi-spool engine where power turbine is not coupled to shaft and face a rapid over speeding. Keeping this thing view, Enalou et al. [62] have developed a transient gas generator turbine model for a three-shaft gas turbine. This model incorporated bleed flow in order to prevent the power turbine from rapid over speeding during load rejection. Blotenberg [97] has tried to reduce the impact of sudden load shedding on the over shooting of power turbine speed of a twin-shaft dry low NOx compliant engine. Generally, dry low NOx combustor contributes in over speeding by storing a huge amount of energy due to enlarged volume. Similarly, a combined transient model of gas turbine, synchronous generator, and fuel governor has been developed by Hung [17] in order to achieve stability during over speeding occurred due to sudden load rejection. Some other contemporary researchers have worked on transient behavior developed by full load and part load operation such as Chacartegui et al. [66] while Badami et al. [48] only considered part load operation. Likewise, Benato et al. [98] have developed and tested a mathematical model for a part load simulation of combined cycle power plant consisting of two gas turbine associated with one air bottoming cycle. 

### 2.4. Secondary Transient Effects

Generally, secondary transient effects consist of thermal dynamics and inlet distortion effects. Whereas thermal dynamics is further divided into four types, i.e., (i) Heat soakage, (ii) turbine’s cooling flow fraction changes, (iii) temperature control sensor’s repose variation, and (iv) turbomachinery clearance changes due to temperature gradients [99]. Transient operation generally involves heat transfer between the working fluid and the engine carcass (metal parts) and this net heat transfer phenomenon is termed as heat soakage. Heat soakage generally occurs in gas turbines in two cases i.e., during hot re-slam and cold start acceleration. Hot re-slam happens when there is a sudden maneuver of engine from high power to idle power range and then instantly jumping back to high power rating without giving enough time to engine carcass to soak heat during low speed. This kind of heat soakage can produce surging in the compressor [100]. Similarly, cold start acceleration happens, when a cold engine is just started at idle speed and suddenly accelerated to maximum power conditions. This may create largest tip clearance changes. Combustors and heat exchangers are more vulnerable to heat soakage because of larger surface area and higher thermal inertia. 

Many researchers considered thermal dynamics effects in their dynamic modeling studies whereas a few scientists have considered inlet distortion effect. Khalid and Hearne [99] developed a very first model of its kind, that address all the aspects of thermal dynamics i.e., heat soakage, fractional variations in turbine’s cooling flow, control sensor temperature response variations and turbomachinery clearance changes, that occur during transient behavior of a turbo fan engine [101]. The substantial effects of these thermal dynamics aspects on the transient behavior of gas turbine, motivated various researchers to include these thermal transients in their control system model for betterment of operational stability. Sometimes transient operation creates various changes in both radial and axial dimensions of turbomachinery components due to variations in thermal and mechanical loading during the operation. Owing to this reason, a relative movement take place between the rotating and stationary parts leading to a thermal growth of engine components. Eventually this thermal growth happens to be responsible for the expansion of the engine metal and variation in clearances occurs. Pilidis and MacCallum [102] studied the transient effect of radial tip and seal clearances in the two spool bypass engine. In another study, they simulated a transient model considering both thermal and mechanical effects. Former effects has been simulated by analyzing the blade tip movement and casing movement while for later case effect, disc, blades and casing thermal growth have been incorporated in the model [103]. However, in order to avoid from severe thermal effects during transient operations, they came up with an idea of selection of an appropriate fuel schedule in another study [104]. Sometimes, these severe thermal effects may lead to reduction of surge margin in the compressor that can create gas turbine failure especially in military aircrafts and fighter jets. This issue has motivated Larjola [105] to develop correction factors for these thermal effects to observe the very effect on surge margin. 

**Figure 6:**
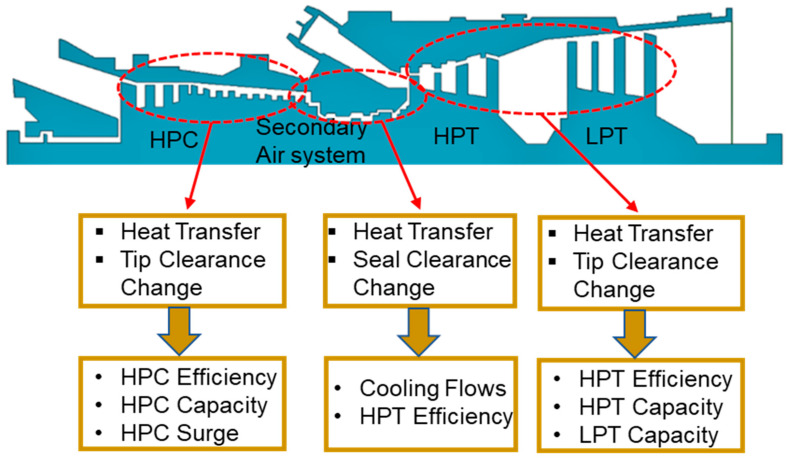
Effects of secondary transients on the performance of the components.

In addition, Nielsen et al. [106] has also conducted a study in order to get insight of the effects of heat transfer on the characteristics of various gas turbine components i.e., compressor, secondary air flow system and turbines as shown in Figure 6. This study also quantified the effects of tip clearance on the surge margin in the compressor section, by developing correction factor for performance maps generation. Merkler et al. [107] have introduced a matrix method and identified the matrix coefficients for modeling of the effects of temperature transients and mechanical stresses on the performance of gas turbine. This method can be utilized for integration with future performance modeling simulation programs. In another study, Merkler and Staudacher [108] have done a comparison of three heat transfer transient modeling methodologies from the literature i.e., replacement structure model (RSM), impulse response model (IRM) and state space model (SSM). Apart from this contemporary researchers such as Giuntini et al. [37] and Vieweg et al. [109] have emphasized on the need of considering thermal stresses during transient model and incorporation of volume dynamics for accurate transient simulations results. Inlet flow distortion and component deterioration has been studied by Novikov [55] by developing a transient aero-thermal model. Although many researchers have worked for thermal transient, but they never worked for industrial gas turbine because military and other aero engine may face rigorous heat transfer due to sharp maneuvers and can face detrimental effects. 

## 3. Methods and Techniques for Transient Models

**Figure 7:**
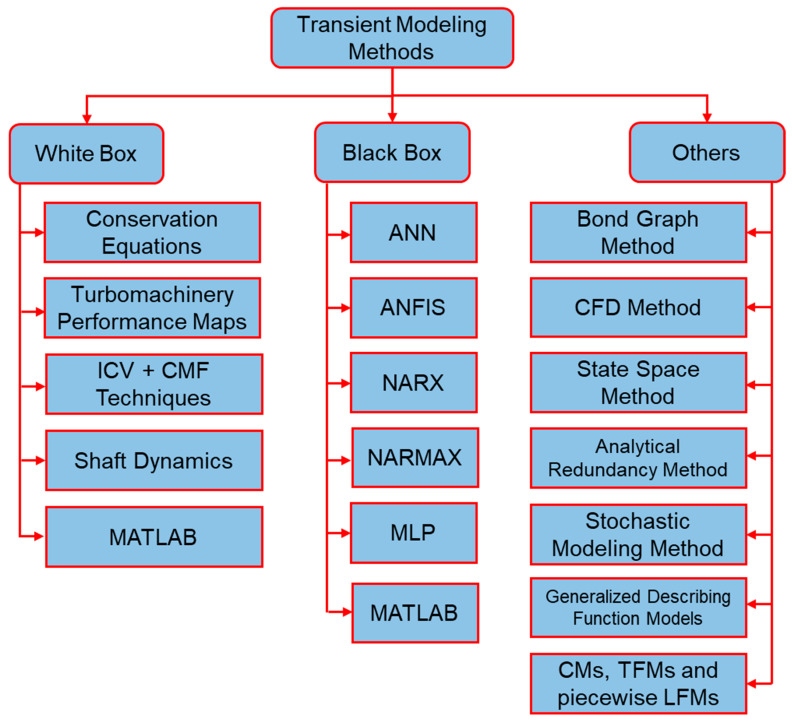
Transient modeling methods adopted in literature.

Generally, transient models for gas turbines are categorized into three types, i.e., white box models, black box models and other models. White box models are also termed as physical models or first principle models because they are based on a profound information about the physics of gas turbine. Moreover, these models are developed by utilizing dynamic mathematical and thermodynamic equation that defines the nonlinearity of the system. These nonlinear equations are simplified by assuming some values as ideal and then applying some linearization techniques using MATLAB simulation environment. Similarly, black box models are also known with another name that is data driven models. These models do not need any information about the physics of the gas turbine, rather they are based on the correlations between the input and output operational data. Usually artificial neural network (ANN) approach is adopted in order to develop black box modeling. A tree diagram representing all the transient modeling methodologies have been illustrated in Figure 7. The detailed discussion about the aforementioned methods is given in the following sections.

### 3.1. White Box Models

A variety of pertinent techniques and methodologies exist in the literature regarding the physical transient modeling methods. Normally, IGTs overall models are based on the individual constituent components’ models such as intake duct, compressor, combustor, turbine and exhaust duct. Physical transient models are normally developed by incorporating ordinary differential equations (ODEs) and some algebraic equations that are formulated after applying physical conservation laws such as mass, momentum and energy for each individual constituent component of IGTs as reported in References [110,111,112]. A variety of approaches exist in the literature to develop accurate transient models; however, the complexity of the methodology depends upon the application and configuration of the target engine. The evolution of the dynamic models started from simplified first principle models to frequency domain and time domain models [71,73,113]. Apart from this, majority of overall gas turbine’s transient models are based on the component performance maps as reported in References [10,35,45,52,71,75] and few models considers the geometrical analysis of the components in order to find the components performance characteristics that is quite difficult approach owing to scarcity of geometrical dimensional data [114]. In order to address the dynamic effects in component level models, quasi-steady state assumptions need to be employed for every constituent component along with corrections, as suggested by Camporeale et al. [71,113]. However, turbomachinery performance maps are not involved as such in very complex models, i.e., models involving the effects of variations of VIGV, VSV, VAN, and inter-stage bleed, on the performance of turbomachinery. Owing to this reason Kim et al. [73] has suggested a stage by stage performance analysis method, by developing one dimensional mathematical equations derived from integral conservation equations. Later on, this study was extended to the startup transient model for heavy duty gas turbines [12] and combined cycle gas turbine [72]. In general, shaft dynamics equations are accounted during transient model development phase. There is another phenomenon that is known as gas dynamics and it involves flow imbalances due to mass accumulation inside different components’ control volumes of IGT [115]. In order to treat these gas dynamics, flow imbalances, two kinds of techniques have been adopted in the literature. The first one is the constant mass flow (CMF) method and second one is the inter-component volume (ICV) method. The CMF method is an iterative method that is based on the initial guess of the component characteristics parameters, i.e., pressure ratio. The flow compatibility is achieved by repeating the initial guesses until the error is minimized to zero by using Newton-Raphson iterative algorithm. Similarly, ICV incorporates component volumes between the interconnected components in order to study the discrepancy in the mass flow. As far as the utilization of these method in already developed transient models is concerned variety of researchers have considered these methods such as Ki et al. [89], Kong et al. [32] and Peretto, and Spina [77] have adopted CMF iterative method along with conservation equations. On the other hand, some researchers such as [43,52,55,76,116] have utilized ICV method along with conservation equations in MATLAB environment. Moreover, in order to avoid form initialization problems due to transient fuel change, Tsoutsanis and Meskin [23] have incorporated both CMF and ICV methods. CMF has been used during steady state iterative component matching to ensure flow and work compatibility while ICV for transient simulation. Similarly, few other studies [9,109,117] have also employed combined CMF and ICV methods in their physical transient models. However, it is very difficult to comment on the usability of both methods as each method holds its own potential significance for different types of scenarios as mentioned in the Reference [116]. 

As far as solution of non-linear partial differential equation (PDEs) and ordinary differential equations (ODEs) involved in the physical models are concerned, a proper numerical technique is required to convert them into a linearized equation for simulation purposes. Various numerical methodologies such as the Newton–Raphson Method [7,10,12,45,71,73,110], Runge–Kutta method [66,73,74,118], Taylor series [119], Euler implicit and explicit numerical solution method [118,120], finite difference method (FDM) [52,66,110,121], linear interpolation method [74], and trapezoidal rule [66,122] have been purposed and utilized by the researchers. However, each method has its own benefits and limitation depending upon the complexity of the mathematical equations involved in transient models.

### 3.2. Black Box Models

**Figure 8:**
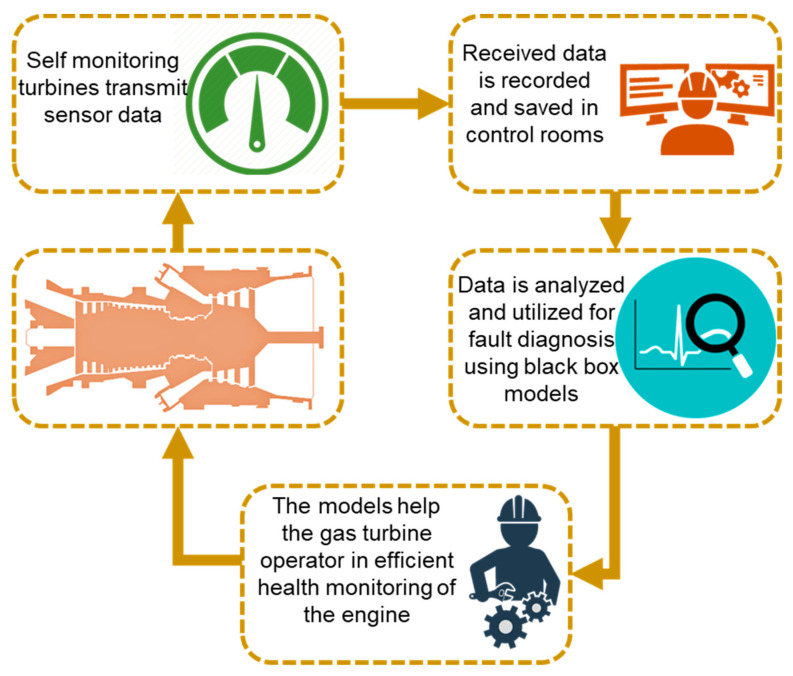
Data driven modeling process cycle.

Industrial revolution 4.0, urges the advancement of the operational technology (OT) with the same pace as the information technology (IT) for enhancing the reliability of the industrial equipment via end to end automation. This automation needs robust and super sensitive sensors technology that can assure extra speed and reliability in various complex machines such as gas turbines. Owing to this fact, General Electric (GE) has planned to equip every mechanical device with high technology sensors because it has been estimated that incorporating these massive amount of sensors in the GE aviation can save 2 billion US dollars per year whereas, the energy sector can save double of the aviation [123]. Considering gas turbine as the potential self-monitoring system; sensors installed in IGT, send the data to control system for further analysis. However, this data provides a real time information about the recent condition of the engine components that help in preventive maintenance and eventually, can decrease unplanned down time. The availability of this sensors’ data has motivated the contemporary researchers to develop algorithms that might help in super-fast and intelligent fault diagnostics and prognostics of IGTs [16]. Hence, the idea of black box models was evolved that are based on only the real time operational data irrespective of the non-linear complex dynamics of the gas turbines systems. A data driven modeling process cycle has been illustrated in Figure 8.

Black box models are usually deemed as branch of artificial intelligence (AI), that revolutionized the computational modeling and simulation of industrial systems. A typical black box model usually establishes a relationship between different variables of the gas turbines obtained from the real time operational data or form the simulations data. In modeling perspective, artificial neural network (ANN) has been implemented widely among various other data driven methods, due to its inherent ability of capturing nonlinear dynamics accurately. Apart from this, ANN encompasses several other approaches such as adaptive network based fuzzy inference system (ANFIS), nonlinear autoregressive with exogenous inputs (NARX), nonlinear autoregressive moving average with exogenous inputs (NARMAX), feed forward multilayer perception (MLP). However, ANN have been used extensively for fault detection and diagnostics of gas turbines in the past. As far as transient model based on ANN is concerned, Asgari et al. [124] is considered as the pioneer of developing transient black box model of heavy duty industrial gas turbines. In this study, physical based model has been developed in MATLAB/Simulink while transient model has been established using the NARX approach by employing same operational data set. Lately, this transient model was expanded to the startup operation of single shaft heavy duty industrial gas turbine being operating in Italy [125]. It can be inferred that, NARX approach can only be utilized for developing models of the engines with available operational data. However, they cannot be utilized for design optimization of the already developed engines. Recently, Mehrpanahi et al. [34] have utilized some neural network (NN) based functions along with time delayed transfer function to develop a start-up transient operation of an IGT using condition monitoring data. However, the prime focus of the study remained toward determination of various shaft characteristics that creates plenty of complexities due to unspecified parameters during startup operation. In his early studies, Mehrpanahi et al. [126] built a semi simplified dynamic black box model for triple shaft industrial gas turbines and addressed the most common problems of the dynamic systems such as time lag of sensors and actuators of gas turbines. Similarly, in another study, Mehrpanahi et al. [35] have integrated black box model with a physical model, in order to develop a grey box transient model for a triple shaft industrial gas turbine. This grey box model proved to be helpful in determining all the possible operating variables at different points during design and off design behavior. Apart from this, this dynamic model is highly precise and speedy in terms of generating the effective variables involved in the system performance. 

### 3.3. Other Models

The bond graph method is another technique to develop transient models for industrial gas turbine. The bond graph technique usually deals the different components of the gas turbines as basic functional units (BFUs), assuming them as lumped elements that help in deducing the dynamic characteristics of the gas turbines through lumped parameters approximations. Recently, Göing et al. [127] utilized a pseudo bond graph method and implemented it in an in-house developed ASTOR program to develop a dynamic model for a turbojet engine. Similarly, Montazeri-Gh et al. [57] have used bond graph approach and hardware in loop (HIL) testing in order to get insightful knowledge of dynamic behavior in industrial gas turbines. Additionally, the same research group has utilized bond graph approach in two other studies as mentioned in References [128,129]. Although it is quite simple method in terms of tracking the nonlinear and complex dynamics of gas turbine, it has some limitations such as it considers the working fluid as ideal gas that is not practical approach. Similarly, in order to develop model, it need geometrical details of gas turbine that are hard to get from manufacturer. 

In addition to the afore-mentioned methods, there are few other methods that have been utilized to develop transient models. For instance, Barbosa et al. [67] have adopted computational fluid dynamics (CFD) approach in order to find the turbomachinery components performance characteristics to be further utilized for transient model. Varadhrajan et al. [130] have developed the dynamic model for using two methods. Firstly, ANN has been utilized in order to estimate the components performance characteristics and secondly, dynamic model has been embodied by using reduced order state space method assuming one dimensional conservation equations. Similarly Kim et al. [88] and Chae et al. [131] have also adopted state space models for transient models. Similarly, Martin et al. [132] has also developed a transient thermodynamic model for a civil aircraft’s turbofan engine using CFD approach. Similarly, Marsilio et al. [133] also used CFD approach. Litchsinder and Levy [22] used novel generalized describing function. On the other hand Kulikov et al. [134] has proposed a linear and non-linear stochastic model identification method for dynamic simulations of gas turbines. Merrington et al. [119] used analytical redundancy method for establishment of transient model for fault detection and diagnostics. Apart from this continuity models (CMs) [135], transfer function models (TFMs) [136] and piece wise linear function models(LFMs) [17] have also been developed over the course of history for analog computers dynamic simulations.

## 4. Transient Model Development Portfolio of IGTs

Overall performance modeling of the gas turbine is based on the modeling of its constituent components, i.e., (1) intake duct, (2) compressor, (3) combustor, (4) turbine, (5) exhaust duct. Component-based modeling subsequently leads to overall performance modeling. Component based modeling is a very accurate and useful tool not only for performance prediction but also it also helps in estimation of overall performance deterioration based on performance degradation of individual components [137]. Unfortunately, realization of performance-based model is somewhat difficult due to unavailability of the component performance data. 

The overall transient behavior of gas turbines that comprise of working fluid and rotating machinery is manifested in terms of conservation equations and motion equations. In order to predict a thorough transient characteristics of gas turbines, unsteady three-dimensional conservation equations may be utilized. However, a simulations model based on unsteady three-dimensional calculations need high fidelity computational endeavors and deemed inefficient. Owing to this issue, several researchers [20,138,139] have proposed unsteady one dimensional calculations that appeared to be more efficient and accurate in terms of capturing the transient characteristics of gas turbine. These studies incorporate spatial discretization in order to derive ODEs from the partial differential equations (PDEs) to simulate dynamics behavior. In this regard a special care is needed for derivation of the equations for compressor, combustor and turbine because in these components the shaft power and force terms cannot be described explicitly in the PDEs. The reason lies in the fact that afore-mentioned components are considered as finite volume while the PDEs are derived from infinitesimal volume analysis. In order to avoid this kind of discrepancy in governing equations, Kim et al. [73] has suggested an integral form conservation equations that is quite easily understandable approach. The final ODEs derived are as follows:

Conservation of mass equation:

(1) $$\int^{\;}\left[\frac{\partial \rho }{\partial t}+\nabla .\left(\rho u\right)\right]dV=0\;$$

Conservation of momentum equation:

(2) $$\int^{\;}\left[\frac{\partial \left(\rho u\right)}{\partial t}+\nabla .\left(\rho uu\right)\right]dV=-\int_{S}pndS+F$$

Energy conservation equation:

(3) $$\int^{\;}\left[\frac{\partial \left(\rho \hat{e}\right)}{\partial t}+\nabla .\left(\rho \hat{e}u\right)\right]dV=\dot{Q}-{\dot{W}}_{s}-{\dot{W}}_{p}$$

Whereas:

(4) $$\hat{e}=e+\frac{1}{2}\left(u.u\right)$$

Further integration of the above mentioned PDEs give simplified ODEs that can be solved by applying initial and boundary conditions. The governing equations shown below can be applied to any component of the gas turbine while considering that component as a finite volume. These set of equations assist in finding the exit parameters of any stage for specific time step as follows:

Mass conservation equation:

(5) $$V\frac{d\rho_{i+1}}{dt}=-{\dot{m}}_{i+1\;}+{\dot{m}}_{i}$$

Momentum conservation equation (F = ma, Newton second law of motion):

(6) $$V\frac{d{\left(\rho u\right)}_{i+1}}{dt}=\;-\left({\dot{m}}_{i+1}u_{i+1}-{\dot{m}}_{i}u_{i}+p_{i+1}A_{i+1}-p_{i}A_{i}\right)+F$$

Energy conservation equation:

$$V\frac{d{\left(\rho H-p\right)}_{i+1}}{dt}=\;-\left({\dot{m}}_{i+1}H_{i+1}-{\dot{m}}_{i}H_{i}\right)+\dot{Q}-{\dot{W}}_{s}$$

Whereas the force (F) and shaft power (${\dot{W}}_{s})\;$ are determined from the following relationships and asterisk symbol is representing the steady state outlet characteristics of a finite control volume for specific time step:

(7) $$F=\left({\dot{m}}_{\left(i+1\right)}^{*}u_{i+1}^{*}-{\dot{m}}_{i}u_{i}+p_{i+1}^{*}A_{i+1}-p_{i}A_{i}\right)$$

(8) $${\dot{W}}_{s}={\dot{m}}_{i}\left(H_{i}-H_{i+1}^{*}\right)$$

### 4.1. Shaft Dynamics

Shaft transients are of paramount importance during transient behavior of IGTs. In general, during transient behavior, shaft generate an extra work output than that is needed to balance the compressor and turbine work output, due to inertia of the shaft and rotating parts attached to the shaft. Apart from this, discrepancy of torque between the rotor shaft and generator load initiates fluctuation in shaft speed. That is why, application of law of conservation of angular momentum becomes indispensable for shaft dynamics modeling [69]. Hence, angular acceleration of the shaft that totally depends upon the moment of inertia (J) of the shaft and the other integrated rotary components, can be represented from the following equation [113]:

(9) $$\frac{d\omega }{dt}=\frac{1}{J\omega }\left[\dot{W_{t}}-\dot{W_{c}}-\dot{W_{f}}-\dot{W_{el}}\right]$$

The above equation, typically represents $\dot{W_{t}}$ as power output produced by turbine, $\dot{W_{c}}$ as power input required by compressor, $\dot{W_{f}}\;$ as power loss due to mechanical friction and $\dot{W_{e}}$, as the power output required by the electric load or generator. If an IGT consists of more than one shafts then, the above-mentioned equation needed to be rearranged in non-dimensional form, to be applied to every shaft individually. The rearranged non dimensional equation is given below:

(10) $$\tau_{I}\frac{dn}{dt}=\frac{N_{d}^{2}}{{\dot{W}}_{u,d}N}\left[\dot{W_{t}}-\dot{W_{c}}-\dot{W_{f}}-\dot{W_{el}}\right]$$

Whereas the characteristics time constant $(\tau_{I}$) can be determined from the following expression,

(11) $$\tau_{I}=\frac{J.\omega_{d}^{2}}{{\dot{W}}_{u,d}}$$

$\omega_{d}$ and ${\dot{W}}_{u,d}$, mentioned in the above expression are angular shaft speed and turbine power output respectively. This kind of formulations helps in achieving accurate simulation results during idle speed of turbine startup. Apart from this, it assures that work output loss, that is determined by loss factor, is a function of the angular speed [71]. 

After an in-depth investigation into the studies related to transient modeling for gas turbine, it became evident that there is no such evidence of estimation of shaft moment of inertia, rather it has been assumed randomly without any justification. Janikovic [140] from Cranfield University has proposed a range for shaft’s moment of inertia that is $J=30-50\;kg.m^{2}$. The polar moment of inertia values used by various scholars is listed in Table 2. Moreover, Kim et al. [64] have proposed a relation in order to find the moment of inertia of the engine based on scaling principles as follows:

(12) $$J_{target,Eng}=\frac{{\left(mr^{2}\right)}_{target,Eng}\;}{{\left(mr^{2}\right)}_{Ref,Eng}}\times J_{Ref,Eng}$$

However, this formulation holds valid only when, weight and diameter of the both, the reference engine and target engine are known. Similarly, the moment of inertia of the refence engine should also be known to get moment of inertia of engine under study. This formula has limitation in terms of geometric data, i.e., weight and diameter, that are hardly available in the public domain. Owing to this problem, most recently, some researchers such as Filinov et al. [141], Kuzmichev et al. [142] Tiemstra [143], and Lolis [144] have conducted some studies in order to estimate the weight and inertia of some turbofan engines. Hence, a research gap still exists in the literature to develop correlations for estimation of shaft moment of inertia for both aero and industrial gas turbines to get accurate transient simulations results.

**Table 2:** Polar Moment of inertia values used in the literature.

Authors	Polar Moment of Inertia (kgm^2^)		Configuration of Engine
	$\mathrm{GG}\;(J_{GGS})$	$\mathrm{PT}(J_{PTS})$	
Gaudet, [145]	0.08	0.05	Twin shaft (Marine)
Janikovic, [140]	30–50	50	Twin shaft (Turbofan)
Novikov, [55]	0.060334	1.3694	Twin shaft (Turboshaft)
Silva, [146]	0.55	0.35	Twin shaft (Turboshaft)
Barbosa et al. [147]	0.0125	-	Single shaft (Turbojet)
Kim et al. [64]	1.14	1.60	Three shaft (Turbofan)
Kim et al. [72]	0.02	-	Single shaft (IGT)

GG = gas generator, PT = power turbine.

### 4.2. Volume Dynamics

During steady state operating conditions, the mass flow entering in specific component is supposed to be equal to outgoing mass flow. However, this assumption does not hold valid in case of transient behavior. For instance, if fuel is increased in the combustion chamber, the turbine entry temperature will be increased. In practice the turbine is assumed to be in chocked conditions having constant inlet and outlet non dimensional mass flows, that is why to ensure this law of conservation of the mass, the pressure of the combustor need to be increased. This pressure rise is due to increased mass flow caused by additional fuel flow. The bigger the volume of the combustor, the greater the pressure rise inside the combustor. This phenomenon is known as volume dynamics, but there are some other names that might be associated with this such as volume packing and gas dynamics etc. [140]. For modeling the transient behavior two kind of methods have been suggested by Fawk and Saravanamuttoo [116], i.e., constant mass flow (CMF) method, and intercomponent volume (ICV) method that are explained as follows.

#### 4.2.1. Constant Mass Flow Method 

This method assumes that the inlet mass flow of a component of gas turbine as equal to outlet mass flow, hence ensuring conservation of mass principle. In order to simulate dynamic simulation, some parameters such as compressor pressure ratio, turbine mass flow and some iterations cycles need to be guessed to help engine quickly shift from one operating state to another [43]. That is why, this method was termed as fast as compared to other method because it considers larger time intervals for iterations that make the iterative process very faster [116]. Apart from this it proved to be useful for dynamic simulation back in the days when the computers were not so fast. Recently, Kurosaki et al. [148] have proposed a new volume dynamics model in order to enhance the computational fidelity by using implicit Euler method for numerical solution of the dynamic system. However, the suggested method takes larger time steps during simulation process. The Jacobian matrix has been computed as a function of corrected shaft speed and input conditions to avoid from the iterations during simulations.

#### 4.2.2. Inter Component Volume (ICV) Method 

**Figure 9:**
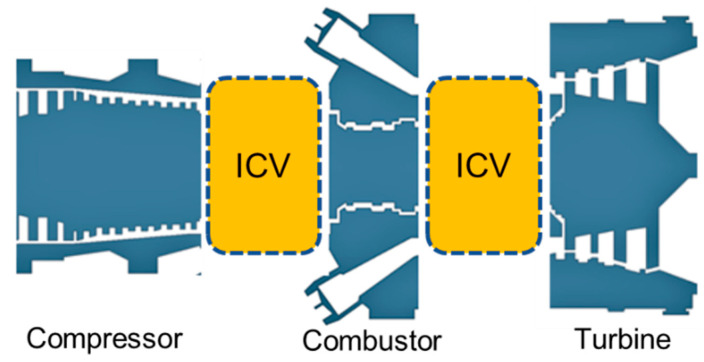
Single shaft IGT with Inter-component volumes.

**Figure 10:**
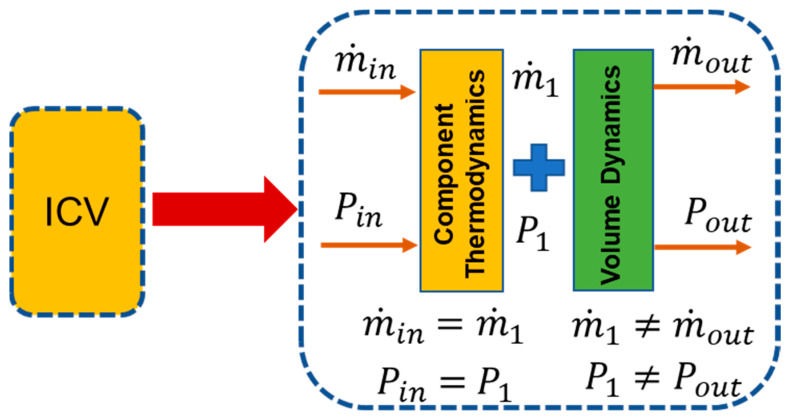
Physics of inter-component volume.

**Figure 11:**
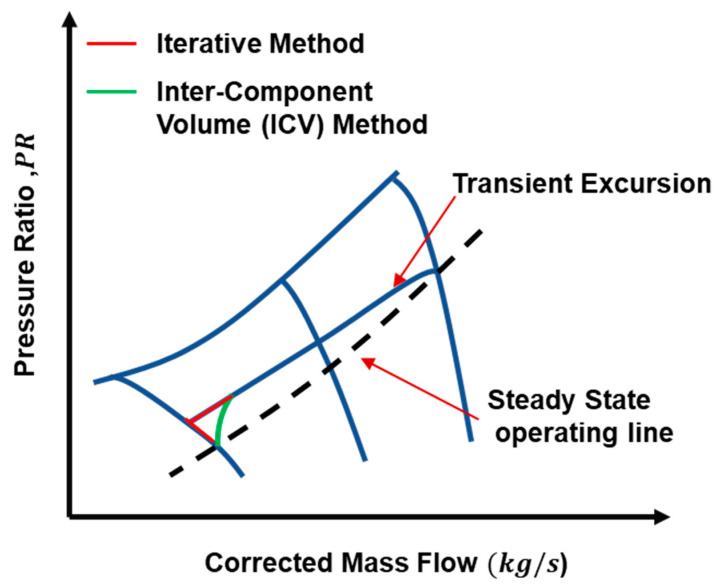
Constant mass flow (CMF) and inter-component volume (ICV) methods trajectories on performance maps.

During transient behavior engine face imbalances in the working fluid’s inlet mass flow and outlet mass flow because mass is not conserved during transient behavior. In general part of the mass of the working fluid is stored inside the control volume of any component as shown in. That is why inter-component volumes are usually introduced between the two interconnected components [117] as shown in Figure 9 and Figure 10. Hence this mass flow mismatch help in estimating the pressure rise at various engine stations. This method proved to be more accurate because it takes very short time interval for computation and need a robust computational efficiency. Hence this method has been preferred over CMF in modern day’s digital simulation models. If we compare both methods, then it becomes evident that both methods give quite similar results, but the difference lies in the operating line trajectory on the compressor maps [116]. For example, during initiation of acceleration, the operating line trajectory is smoother by adding inter component volumes while trajectory takes sharp maneuver with CMF method as shown in Figure 11.

### 4.3. Inlet and Exhaust Duct Modeling

During every model development case, application of one-dimensional conservation equation is deemed imperative in order to ensure that mass, momentum and energy are conserved in each constituent component. The first and foremost component in IGT modeling is intake duct. However, in the conservation equation, the shaft power and heat transfer in assumed to be negligible (i.e., ${\dot{W}}_{s}\;$=$\;\dot{Q}\;$= 0). Hence, the total pressure loss during transient behavior, inside the intake and exhaust duct can be calculated from the following equation, as suggested in Reference [73,114].

(13) $$\frac{\left(\frac{\Delta P}{P_{in}}\right)}{{\left(\frac{\Delta P}{P_{in}}\right)}_{d}}=\;\frac{{\left(\dot{m}\frac{\sqrt{T}}{P}\right)}_{in}^{2}}{{\left(\dot{m}\frac{\sqrt{T}}{P}\right)}_{in,d}^{2}}\times \frac{R}{R_{d}}$$

### 4.4. Compressor Modeling

The overall performance of an axial compressors is normally measured and quantified by performance maps that characterize the performance and behavior of a compressor at different operating conditions. The most important performance parameters that are the part of a compressor map are mass flow rate, pressure ratio, efficiency and speed of the compressor [149]. In general, these maps can be generated by empirical data or by precise prediction of geometric properties of constituent components, i.e., intake duct, compressor, combustor, turbine and outlet duct [150]. The performance of compressor is visualized by general equation of compressor performance in terms of non-dimensional parameters:

(14) $$\frac{\tau_{c}}{d_{2}^{2}P_{01}}=\;\frac{1}{2\pi }\frac{1}{\eta_{c}}{\left(\frac{d_{2}N}{\sqrt{C_{Pa}T_{01}}}\right)}^{-1}\;\frac{m_{a}\;\sqrt{C_{Pa}T_{01}}}{d_{2}^{2}P_{01}}\;\;\left[{\left(\frac{P_{02}}{P_{01}}\right)}^{\frac{\left(\gamma_{a}-1\right)}{\gamma_{a}}}-1\right]$$

Equation to find compressor power:

(15) $$W_{c}=d_{2}^{2}P_{01}\sqrt{C_{Pa}T_{01}}\;\frac{m_{a}\;\sqrt{C_{Pa}T_{01}}}{d_{2}^{2}P_{01}}\frac{1}{\eta_{c}}\left[{\left(\frac{P_{02}}{P_{01}}\right)}^{\frac{\left(\gamma_{a}-1\right)}{\gamma_{a}}}-1\right]$$

If two non-dimensional parameters are known, then other remaining parameters can be determined easily. All the above equations can be simplified by inserting the values of non- dimensional parameters, picked up from the compressor map. Hence, the non-dimensional parameters that are necessary for compressor modeling are (i) mass flow (ii) speed (iii) Torque (iv) pressure ratio (v) efficiency (vi) temperature and (vii) pressure.

Variety of compressor modeling techniques have been adopted in the literature, i.e., (i) scaling method, (ii) sequential stage stacking method, (iii) modified stage stacking method, and (iv) blade element method. A critical review of these methods have been listed in the Table 3.

### 4.5. Combustor Modeling

Combustion chamber or combustor is a device that is deemed to produce heat input by burning some hydrocarbon fuel for generation of power output in the gas turbine engine. Combustion chamber draws the air from the compressor and then send it to the turbine at an elevated temperature. Eventually the combustion gases are then mixed with the residual air to reach up to a designated turbine inlet temperature (TIT). Generally, the combustion chambers are categorized into three major types, i.e., (i) tubular, (ii) tubo-annular and (iii) annular [4]. Every gas turbine’s combustor is supposed to have three combustion process inherent features: (1) a primary zone, that is responsible for burning the fuel and exploiting heat out of it; (2) an intermediate zone, where combustion process is ensured to be accomplished by introducing additional air; and (3) the dilution zone that is capable of reducing exit gas temperature by introducing remaining air [122].

The pressure loss in combustion chamber may be categorized in two types: (i) cold loss, and (ii) hot loss. The cold loss may occur as a resistance of the combustor components against flow of air and sometimes due to high level of turbulence may extract some energy from the air entering the combustor thus giving a stagnation pressure loss across the combustor. This loss is proportional to the square of velocity. Similarly, hot loss is associated with the addition of heat. Because heat increase may decrease the density, which in turn increases the velocity. This phenomenon is known as *Raleigh flow* [80].

As far as the modeling of the combustion chamber is considered, combustion chamber is assumed to be energy accumulator, that is why mass conservation equations are not included as such during modeling. However, temperature and pressure inside the combustor are assumed to be uniform and equal to exit temperature and pressure. The input parameters that are required for combustor modeling are mass flow, temperature and pressure at compressor’s discharge, and fuel flow and its composition. Similarly, the output parameters at downstream of the compressor are, mass flow, temperature, pressure, enthalpy, and gaseous mixture composition. Hence, the mathematical formulation that represent combustor dynamics has been shown in form energy balance equation as follows [113]:

(16) $$\tau_{cc}\frac{dT_{out}}{dt}=\;\frac{g_{in}h_{in}+g_{cc}\left(h_{b}+\eta_{cc}LHV\right)-g_{out}h_{out}}{g_{out}c_{p_{out}}}$$

The LHV depends on the composition of the fuel used in gas turbine. Whereas, time constant $\tau_{cc}$, can be determined from the following expression:

(17) $$\tau_{cc}=\frac{M_{cc}}{Kg_{out}}$$

$M_{cc}$, is normally total mass accumulated inside the combustor during each time step and it depends upon outlet pressure, temperature, and fuel composition that can be calculated from ideal gas equation assuming complete combustion. Apart from this pressure loss occurring inside the combustor during transient operation can be corrected by using the following formula as suggested by Kim et al. [73]:

(18) $$\frac{\left(\frac{\Delta P}{P_{in}}\right)}{{\left(\frac{\Delta P}{P_{in}}\right)}_{d}}=\;\frac{{\left(\dot{m}\frac{\sqrt{T}}{P}\right)}_{in}^{2}}{{\left(\dot{m}\frac{\sqrt{T}}{P}\right)}_{in,d}^{2}}\times \frac{R}{R_{d}}$$

### 4.6. Turbine Modeling Methods

Similarly to the compressor, turbine characteristics can be demonstrated by turbine maps. The non-dimensional parameters needed to be picked up from turbine maps are same as compressor, i.e.,: (i) mass flow, (ii) speed, (iii) torque, (iv) pressure ratio, (v) efficiency, (vi) temperature, and (vii) pressure. The turbine performance maps are responsible for overall turbine performance characteristics. If two non-dimensional parameters are known, then the rest of the values can be found from performance. Finally, turbine modeling is completed by inserting the non-dimensional values chosen from turbine map in the equation of overall turbine performance given below:

(19) $$\frac{\tau_{c}}{d_{2}^{2}P_{03}}=\;\frac{1}{2\pi }\eta_{t}{\left(\frac{d_{2}N}{\sqrt{C_{Pg}T_{03}}}\right)}^{-1}\;\frac{m_{g}\;\sqrt{C_{Pg}T_{03}}}{d_{2}^{2}P_{03}}\;\;\left[{\left(\frac{P_{04}}{P_{03}}\right)}^{\frac{\left(\gamma_{g}-1\right)}{\gamma_{a}}}-1\right]$$

(20) $$W_{t}=d_{2}^{2}P_{03}\sqrt{C_{Pa}T_{03}}\;\frac{m_{g}\;\sqrt{C_{Pg}T_{03}}}{d_{2}^{2}P_{03}}\eta_{t}\left[{\left(\frac{P_{04}}{P_{03}}\right)}^{\frac{\left(\gamma_{g}-1\right)}{\gamma_{a}}}-1\right]$$

As far as turbine modeling is concerned, variety of researchers have employed various techniques like turbine performance maps [9,10,32,33,35,52,61,74,159], Stodola ellipse equation [7,12,66,71,73,90,121,160,161,162], choking equation [45], Flugel formula [40,163,164,165,166,167,168] and blade element method [45,73,75,111]. If turbine performance is not available, then the turbine off design model is designed by the choking equation. The design points of modern gas turbines are close to a choked condition. Therefore, this method is feasible for industrial gas turbines as long as the turbine expansion ratio does not deviate much from the design value [45]. A brief comparison of some turbine modeling methodologies is listed in Table 4 given below.

## 5. Control Strategies for Dynamic Operations

**Figure 12:**
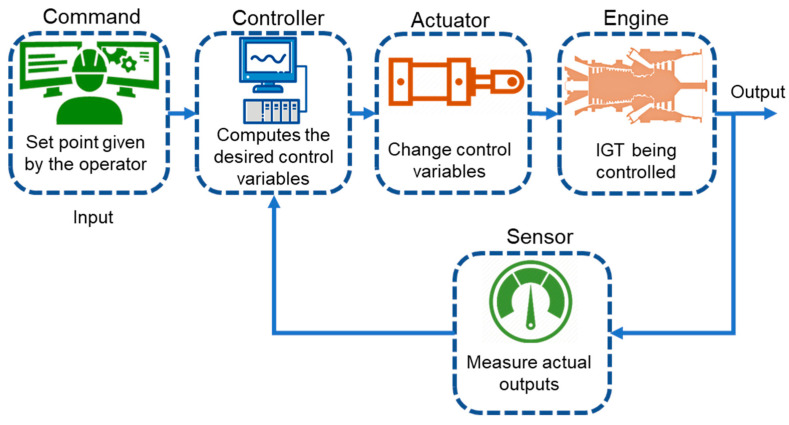
Components of feedback control system.

Closed loop control strategy based on feedback control action is typically utilized in IGTs. The working principle of closed loop schemes is based on the actuating signal that is comprised of the difference between the input and output signals. Eventually, this actuating signal is fed into the controller in order to reduce the error between the demanded and actual value until the desired output is obtained. The feedback control system normally consists of four major entities i.e., controller, actuator, engine, and sensor. Figure 12 shows the complete sequence of control system. The controller in the figure act as a brain of the complete control architecture, because it computes the desired value of control variable by reducing error between the signals coming from measurement sensor and commanded by gas turbine operator. After this, controller decides proper control orders based on certain embedded control algorithm for actuation and regulation of the gas turbine engine. The more advance controller possesses the inherent capability of storing the engine’s overall health data in their embedded numerical models, and hence this data can be further utilized for intelligent fault diagnostics and prognostics. 

### 5.1. Simplified PID Control Scheme

**Figure 13:**
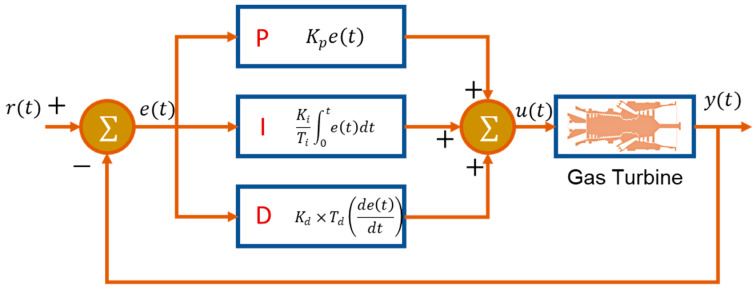
Proportional-integral-derivative (PID) controller for gas turbines.

IGTs used for power generation purpose usually face part load conditions that may lead a complex dynamic behavior. This dynamic behavior may be responsible for abrupt increase in turbine exhaust temperature (TET), shaft rotational speed and surging. That is why there is always need for designing a robust and efficient automatic control system that might ensure overall stability of operational parameters of the engine. Several kinds of control system techniques exist in the literature based on the nature of the dynamic behavior, i.e., startup, load change and shutdown. However, a control system that is used for an engine facing the fluctuation of output power due to variation in electric load is known as load following control. In general, startup and load following operation demand different types of control strategies due a peculiar and more complex nature of startup sequence of IGTs. Rowen [169] gave idea of the very first simplified control system for heavy duty IGTs. This model was capable of controlling speed, temperature, acceleration and fuel limits based on feedback proportional-integral-derivative (PID) control scheme as shown in Figure 13. The simplified mathematical representation of the PID controller is given below:

(21) $$u\left(t\right)=K_{p}e\left(t\right)+\frac{K_{i}}{T_{i}}\int_{0}^{t}e\left(t\right)dt+K_{d}\;\times T_{d}\left(\frac{de\left(t\right)}{dt}\right)$$

In general, two kinds of control systems were used in Rowen’s model, i.e., droop governor and isochronous governor. Droop governor considers only the proportional controller while isochronous needs proportional-integral (PI) in order to minimize the error in demanded speed and actual speed. However, proportional controller involves a residual and recurrent steady state error that cannot be reduced to zero. That is why integral controllers need to be integrated with proportional to avoid this problem. Involvement of integral controller creates overshoot in the signals during transient operation due to shaft inertia. Later on, Kim et al. [73] used this model by modifying control gains for load following dynamic performance study of heavy duty gas turbines. These kinds of PID control schemes have also been used in transient modeling of CCPPs [7]. Chacartegui et al. [66] have done some retrofitting in the simple PID controller and developed two different control schemes, i.e., optimal LQG controller with cascade PI and parallel open loop tuning, and open loop PI controller with serial look up table. In this study, both control schemes showed promising results during steady state off design operation, while open loop control scheme depicted very robust control during dynamic behavior. 

### 5.2. Model Based Control Schemes 

Wang et al. [170] introduced a variation in the conventional PID scheme by integrating self-tuning back propagation neural network with an adaptive PID controller for TET of a micro gas turbine. However, the control schemes involving PID controller are not proficient in presence of processes disturbances due to unavailability of proper controller gain values. In order to avoid from this problem, Nelson and Lakany [171] suggested a new technique fuzzy logic control (FLC) for TET control of IGTs. Moreover, current penetration of renewable energy resources in global electricity market and integration of renewables with IGTs as hybrid systems, demands some cutting-edge advancements in the design of IGTs as well as in the control systems. Hence, model-based control techniques such as sliding mode control (SMC), Feedback linearization (FBL) approach and model predictive control (MPC) have sought the attention of power systems control designer. As sequel to the above mentioned techniques, Panda and Bandyopadhyay [172] and Bonfiglio et al. [173] have suggested a nonlinear control system known as SMC that can accurately capture the nonlinear dynamics based on variable structure control which responds to any dynamic variation instantly. Additionally, SMC are the state of the art, robust and efficient electronic type controllers as compared to traditional hydromechanical controllers, providing higher controllers gains and reducing overshoots and undershoots during transient operations. The most significant problem that occur during transient behavior is that variation of load creates abrupt fluctuations in the temperature of combustion chambers. However, the conventional control systems need the measurements of overall states of system including combustor temperature that is hard to be measured. In order to overcome this problem, recently, Bonfiglio et al. [174] developed FBL technique that is independent of the combustion temperature and give smooth and quick response as compared to conventional PID controller. Another technique that has gained attention in the hybrid power plants is MPC scheme and has been used by Ferrari [175] for hybrid solid oxide fuel cell systems. Similarly, Menon et al. [176] have used this technique for poly-generation systems. So far single loop control strategies have been developed and used for IGTs. The single loop control strategies cannot meet the needs of multi-shaft engine due to complex operational envelope. Hence, Zhang et al. [177] suggested a multiloop control system that can ensure an effective and robust control during load rejection of load following operation of a three shaft IGT. Similarly, Wu and Zhu [178] have also designed a multiloop control scheme for a hybrid system of SOFC and micro gas turbine.

### 5.3. Fuel Flow Actuation

**Figure 14:**
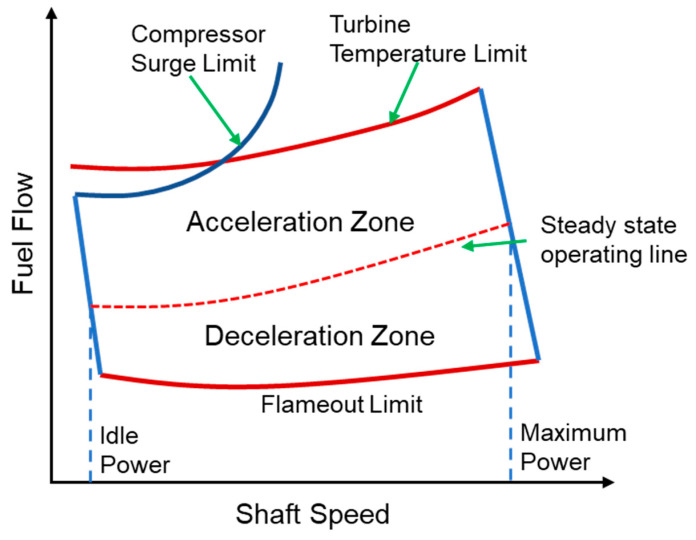
Fuel flow schedule for safe operation.

Actuator play an important role in controlling the engine’s system operations based on the commands given by the controller. Normally, IGT’s actuating system consist three categories, i.e., Fuel control actuators, position control actuators and air flow actuators. Fuel control actuators are typically responsible for actuating the fuel metering valves that are very critical during transient operations. Similarly, position control actuators help in controlling VIGVs and VSVs while air flow actuators are responsible for actuating VBVs in order to prevent the engine from surging and choking during part load operation. As far as, the control system during load following transient operation is concerned, engine come across two kinds of control scenarios, i.e., prime control, and protective control [179]. Prime control support in synchronizing the shaft speed with that of electric generator during fluctuation in power due to abrupt load change. Whereas, protective control is responsible for safe operation of gas turbine and helps in avoiding form serve torque variations due to load change. During overshooting in speed and over temperature phenomena, protective control tends to decelerate engine from rated speed to the idle speed. However, during sudden acceleration, abrupt fuel increase may lead to over temperature that can damage the turbine blades. At this situation control systems opt to ensure safe operation without any catastrophe. Owing to this reason protective control wins over the prime control. In this scenario, a fuel schedule with proper control limits need to be adopted that might ensure a safe operation [180]. A generalized fuel schedule with its control has been shown in the Figure 14.

**Figure 15:**
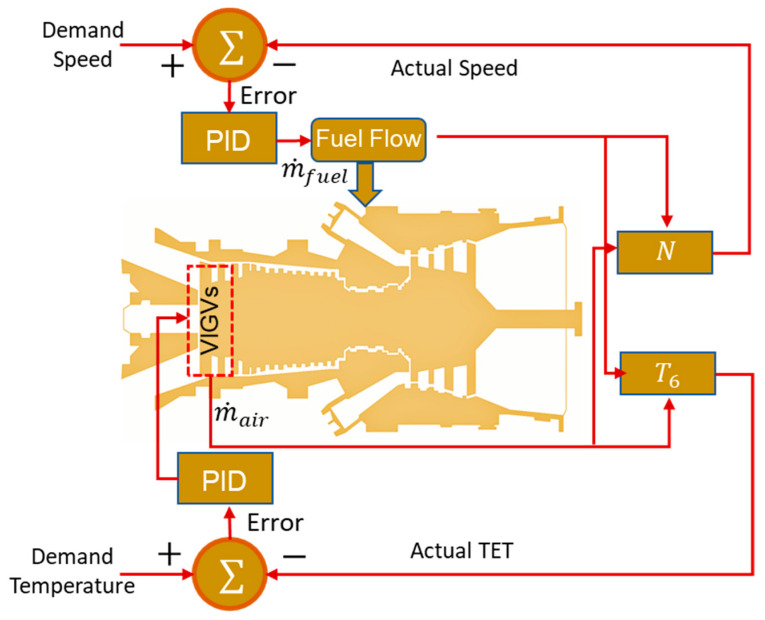
Simultaneous control of speed and turbine exhaust temperature (TET).

During load following transient operation, the fundamental task of the controller is to maintain the speed and turbine exhaust temperature (TET), i.e., *T*_6_, of gas turbine to a specified limit in order to avoid for failures due to thermal and mechanical stresses. These two parameters are controlled by actuating the fuel valve and VIGVs simultaneously in order to keep the fuel and air mass flow at a designated range. A schematic of a PID controller has been shown in the Figure 15 that is representing a simultaneous control of speed and TET by actuating fuel flow and VIGVs. 

### 5.4. VIGV Actuation

Industrial gas turbines are associated with CCPPs nowadays. Since IGTs must work on part load, and hence at the reduced speed. At lower speeds, the reduction in axial velocity is sometimes, surpassed the reduction in the spool speed. This phenomenon creates a flow separation from the blade passage due to increased incidence angle to the blade. Hence this flow separation develops a stall (subsequently surge) in the early stages of the compressor while the rear stages are choked [181]. Surge can be controlled by multi-spooling, variable bleed and variable geometry features, i.e., VIGVs and VSVs. In fact, VIGVs and VSVs helps in reducing the large incidence angle at rotor front stages during sudden fall of speed [182]. Apart from this, these prove to be useful in increasing the surge margin by skewing the angle at a specified spool speed (high or low) guessed by the controller. However, at higher speeds the operation is converse of the low speed, i.e., at high speed the front stages are choked while the rear stages are surged. In this scenario, to avoid choking, the VIGV angle is re-staggered in order to increase the mass flow and pressure ratio by increasing the sectional flow area of the compressor and hence increase the surge margin [182,183,184,185].

**Figure 16:**
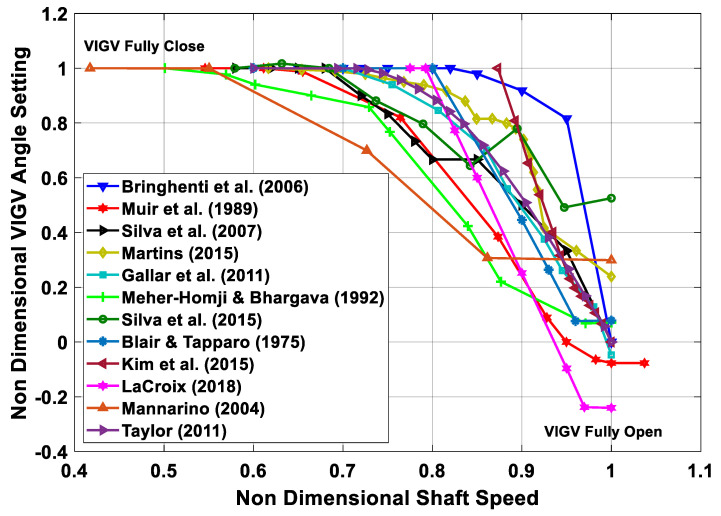
Literature based comparison of various VIGV schedules as a function of shaft speed.

**Figure 17:**
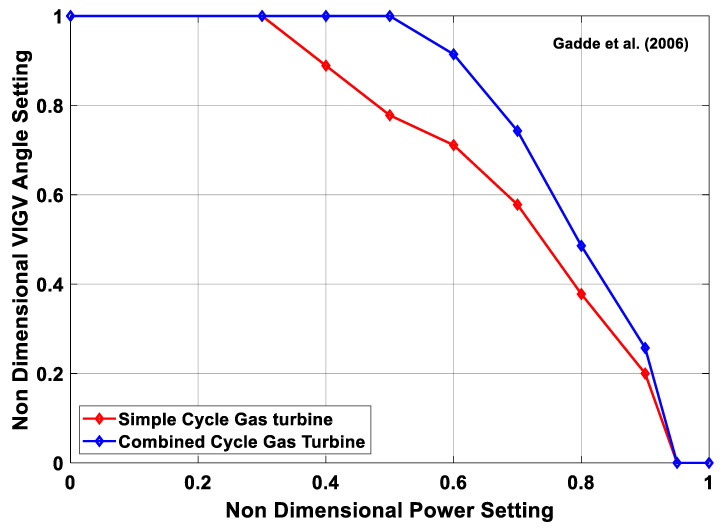
VIGV schedule as a function of power setting.

Generally, the variable geometry compressors are equipped with VIGVs in the beginning followed by 1st rotor and then subsequently VSVs on the following first three stages. These VSVs relate to the single actuator via a common link in order to assure simplicity and avoid from weight and cost problems. However, these can work independently via different actuators [154]. VIGV/VSV and variable bleed schedules are needed to get an optimum surge margin at part load operation of CCPPs [186]. These schedules are represented in forms of maps that comprised of VIGV/VSV angle as a function of compressor’s aerodynamic speed. In practice the VIGV/VSV angles settings are found from rig tests where several kind possible combinations of VSV setting are compared and tested by trial and error method. A comparison of variable geometry schedules from Bringhenti et al. [70], Muir et al. [137], Silva et al. [69], Martins [187], Gallar et al. [182], Kim et al. [64], Mehr-Homji and Bhargava [39], Silva et al. [188], Mannarino [189], Blair and Tapparo [190], and LaCroix [191] has been represented in the Figure 16. This set of VIGV schedules demonstrate that VIGV angle is staggering as a function of shaft speed whereas Gadde et al. [192] proposed another VIGV schedule (see Figure 17) in which VIGV angles are staggering as a function power or load setting, hence making it a suitable fit for single shaft IGT that can be employed for power generation applications. However, a comprehensive VIGV selection framework is discussed in the following section. This detailed collection of different VIGV schedules from various research articles, books, and reports, serves as a database for future researchers in efficient and accurate modeling of variable geometry IGT. Any future researcher or gas turbine operation and maintenance personnel may choose a best schedule according to demand in order to achieve an optimum performance of IGT. 

#### 5.4.1. VIGV Schedule Selection Framework

**Figure 18:**
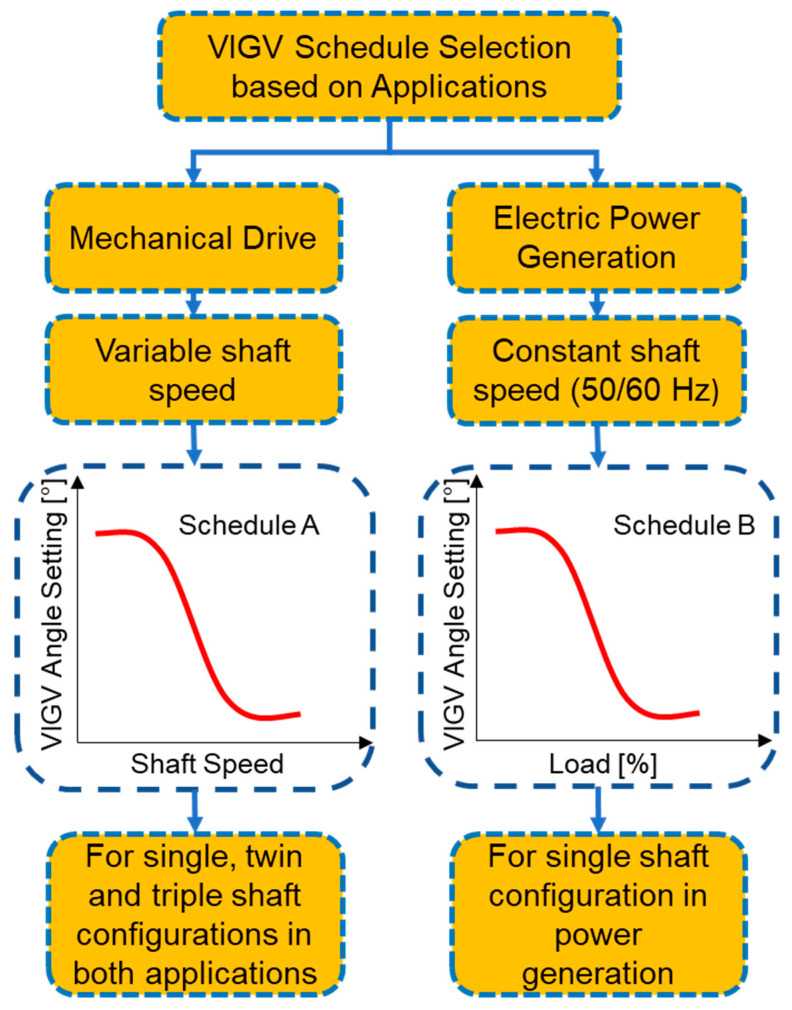
Selection framework of VIGV schedules for different configuration of engines.

The above discussed VIGV scheduled has been segregated into two types, i.e., VIGV schedule (A) and VIGV schedule (B) based on the type of IGTs applications as shown in Figure 18. During mechanical drive application, the shaft speed of the engine is variable, however, requiring a VIGV angle variation as a function of shaft speed. Owing to this fact, VIGV schedule (A) is applicable for all configuration of engine, i.e., single, twin and triple shafts, for both mechanical drive electric generation purposes. On the contrary, during electric power generation the shaft frequency should be equal to the generator frequency (50/60 Hz) in order to meet the power transmission grid requirement. Therefore, the VIGV schedule having VIGV angles varying as a function of shaft speed is no more applicable. Hence, another schedule, i.e., VIGV schedule (B) is utilized. In this schedule the VIGV angles are modulated as a function of load or power setting. Due to frequency limitation this schedule is only applicable to single shaft gas turbines for power generation application as single shaft gas turbine’s shaft speed remains constant though out the operation on full load. Hence, the VIGV schedule selection framework illustrated in Figure 18, assists in sorting out a best VIGV schedule on the basis of engine configuration and application type.

#### 5.4.2. VIGV Modulation Correction Factors

The performance of the turbomachinery components (compressor and turbine) is normally represented in the form of generalized performance maps, as suggested by various authors mentioned in the compressor modeling sections. However, if turbomachinery components are equipped with variable geometry features then, generalized performance maps are not valid. Hence, some corrections factors are employed in order to shift the fixed geometry characteristics to variable geometry characteristics. Correction factors usually quantify the effect of VIGV angle change on two important parameters, i.e., mass flow and isentropic efficiency. In this regard, few correction factors have been proposed by researchers in Table 5:

Similarly, for turbine Δβ is the variable area nozzle angle, that is supposed to be zero at design conditions and the correction equations are given below:

(22) $$W_{corr}=W_{corr}{,}_{map}\left(1-\frac{c_{4}\Delta \theta_{VAN}}{100}\right)$$

(23) $$\eta =\eta_{map}\left(1-\frac{c_{5}\Delta \theta_{VAN}{}^{2}}{100}\right)$$

### 5.5. Variable Bleed Actuation

**Figure 19:**
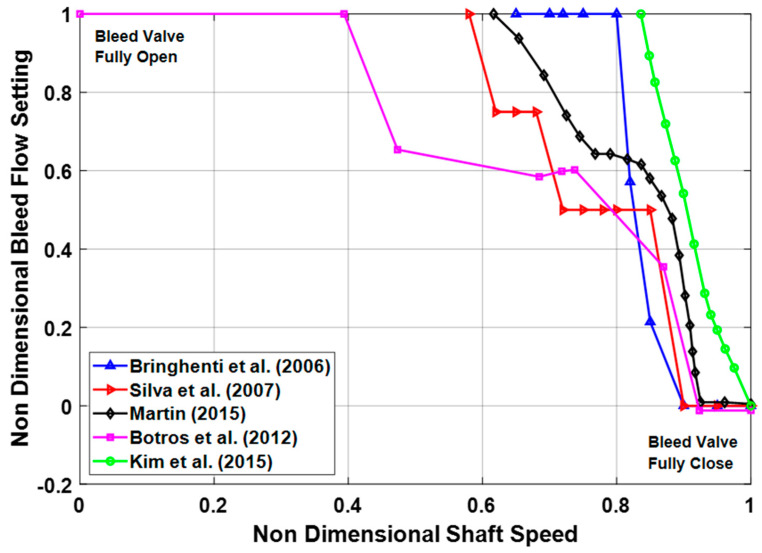
Comparison of variable bleed schedule of various researchers.

The fundamental purpose of the variable bleed is to support the variable geometry system of compressor at lower speeds since at higher speeds the flow is controlled by VIGVs and VSVs. However, at lower speeds the variable geometry angle is too much closed that it is unable to be further closed. That is why, there is a need for a second passage for mass flow reduction and that is what known as variable bleed valve (VBV). This phenomenon is best represented in Figure 19, where VBV is completely closed at higher speeds while fully open at lower speeds. Moreover it helps in managing the primary flow of air entering the HPC, efficiency improvement, surge margin enhancement and regulation of fuel flow during combustion [187]. The trend of VBV schedule is converse of the VIGV schedules as can be observed from illustration. The comparison for variable bleed schedules of Botros et al. [198], Kim et al. [64], Martins [187] Silva et al. [69], and Bringhenti [70] has been represented as a function of corrected shaft speed in the Figure 19. Among these VBV schedules, one can choose the best schedule that might aid in improving the efficiency and surge margin stability. 

The influence of the compressor bleed on the performance of gas turbine is quite significant. For this reason, correct estimation of bleed air mass flow rate (${\dot{m}}_{bleed}$) is necessary because it depends upon the opening of bleed valve that is accustomed to low pressure compressor speed. In this regard Botros et al. [198] has utilized an equation to estimate $({\dot{m}}_{bleed}$) as follows:

(24) $${\dot{m}}_{bleed}=1.1\times C_{g}\sqrt{\rho_{dis}P_{dis}}\;\sin\left(\theta \right)$$

In the above-mentioned equation, $\rho_{dis}$ and $P_{dis}$ are representing density and pressure of the working fluid at compressor’s discharge. Whereas sin(θ) indicates the choking of compressor, i.e., (sin(θ)=1) is assumed for fully choked compressor. However, $C_{g}$ quantifies the extent of bleed valve opening, and its expression is as follows:

(25) $$C_{g}=C_{1}{\left(VBV\right)}_{openfraction}\times C_{v,Max}$$

Here, $C_{1}$ is constant that need to be assumed while ${\left(VBV\right)}_{openfraction}$, indicates variable bleed valve open fraction that can be determined from the variable bleed schedule as shown in Figure 16. Similarly, $C_{v,Max}$ represents the value when bleed valve is fully open, and it can be assumed as $C_{v,Max}=156$.

## 6. Software Tools for Transient Modeling

Gas turbine simulation programs are generally built in order to get acquaintance with performance and operation of the overall system. In this regard, GT simulation programs gained attention by variety of stakeholders such as GT original equipment manufacturers (OEMs), GT operators and academic research community. OEMs usually utilize these simulation program for preliminary design and development of engines whereas these simulation tools are also very helpful for GT operators in engine condition monitoring, predictive maintenance, fault diagnostics, and prediction of remaining useful life of the engine. A variety of software simulation programs have been developed to simulate the steady state and transient operation of gas turbines. However, flexibility, reliability, robustness and user-friendliness features of the simulation program hold paramount importance to the date. The development of simulation programs started back in the middle of 20th century, but unfortunately, the personal computers were analog in that era. After the penetration of digital computers in the global markets, different software were developed that assisted the design engineers and gas turbine operators [199]. These software platforms are typically divided into two categories, i.e., zero dimensional and multi-dimensional that are explained in the following sections. 

### 6.1. Zero-Dimensional Simulation Programs

Zero-dimensional simulation programs are the software environments that provide information about gas conditions at every station of gas turbine, but that do not deal with any physical dimensions of the engine. The beginning of gas turbine zero dimensional transient simulation code started with DYNGEN simulation program that was developed by Sellers and Daneile [200] from NASA. As a matter of fact, DYNGEN is the extended version of the GENENG simulation code that was developed by Koeing and Fishbach [201]. However, this simulation program could merely simulate the steady state that motivated Sellers and Daneile to develop a software for dynamic simulation by integrating dynamic equations in the already available program. In addition to that, Geyser et al. [202] applied some modification to DYNGEN by appending a linearization tool in order to capture nonlinear dynamic behavior via different matrices and named it as DYNABCD. This program had the inherent capability of simulating both steady state and transient behavior of turbojet and turbofan engines. This model was typically based on analytical method using conservation equations. However, researchers at aerospace department Delft University of Technology (TU Delft), Netherlands detected some bugs and instabilities in DYNGEN simulation program. Lately, in 1986, Technical university of Delft in collaboration with National Aerospace Laboratory (NLR), went through modification related to iteration process and graphical user inter face (GUI) and finally Gas Turbine Simulation Program (GSP) [203] was developed in 1996, from the DYNGEN code. Now, GSP is a commercially available object-oriented simulation program that is considered as more flexible and user friendly due to drag and drop GUI. MacMillan [204], developed a zero dimensional simulation program TURBOMATCH based on FORTRAN environment. This program seemed flexible in terms of modifying thermodynamic parameters at that time. Palmer and Cheng-zohng [205] extended the previous work to another simulation program TURBOTRANS, that was capable of performing steady state and transient simulation but control system simulation were also included in it and it was claimed as accurate analytical model. Until now, Cranfield University kept updating this program through the postgraduate researchers. Two scientists named Sadler and Melcher [206], from NASA Lewis research center developed DEAN simulation program that was able to model overall turbofan engine and its sub systems with quite ease. It gave the opportunity to analyze the data in more details via interactive graphics. Poole et al. [207] build a simulation program in C++ environment that could simulate only steady state simulation. However, it was efficient in design of fuel controller for industrial gas turbine. 

The programs developed so far were using turbomachinery performance maps. Although components maps are a better way of approximating engine behavior, they cannot provide basic input parameters, i.e., aero-thermodynamic conditions and turbomachinery blade rows, that are needed during engine control system design. Because these parameters, help in active surge control by staggering the blade rows. Owing to this reason, Schobeiri [208,209,210] and Schobeiri and Haselbacher [211] built a modularly structured simulation program COTRAN for transient modeling and simulation. This program covered the limitation of previous programs due to performance maps, by doing a row by row analysis of expansion process using stage characteristics. This program still has the limitation because it can be used only for single shaft gas turbine. Keeping this in view, Schobeiri et al. [138] extended the code to a new simulation program named GETRAN, that not only helped in simulating the various dynamic behaviors of multi-spool aero engines, but also had the ability of simulating power generation IGTs. In fact, this was the first simulation program that was able to perform transient simulations for variable geometry gas turbines because turbomachinery models were based on adiabatic and diabetic row by row calculation that accounted for blade geometry and turbomachinery cascades [111]. Recently, Schobeiri [58] has done retrofitting in the GETRAN code in order to observe the effect of staggering vane angle of the turbine on the overall dynamic performance and safety of Brown Boveri GT-9, IGT engine. The variation in turbine vane angle as function of time played a vital role in surge prevention. Similarly, two researchers Hale and Davis [212] from Arnold Engineering Development Centre (AEDC), given DYNTECC simulation code that was able to simulate and analyze the dynamic events such as post stall behavior and predict the instability in the axial compressor. This one-dimensional code was based on stage by stage turbomachinery mathematical equations and source terms, i.e., bleed mass flow, blade force and shaft power. As a sequel to this code, Garrard [213] enhanced this simulation code to another program named ATEC. This program can simulate both transient and dynamic events such as compressor stall and combustor blow out. Apart from this it also incorporated combustor modeling schemes and turbine modeling scheme. It also works on the same principle of representation of compression process through stage by stage calculations, as DYNTECC. However, this program takes very large time steps during steady state and transient operation and reduce the calculation time because of implicit and explicit equations solvers. Meanwhile, an engineer named Dr. Kurzke from MTU Aero Engines Germany, developed a simulation program GasTurb in last decade of twentieth century [214,215]. GasTurb was developed in an object-oriented language environment Borland Delphi, to create a user-friendly interface that give choice to user in selection of desired configuration among the configurations available in the simulation tool. Although this software is flexible and user-friendly owing to hidden nature of the code, it can only simulate the configuration available inside the code, i.e., one cannot develop user defined configuration. In this context, GSP is considered the most robust and flexible package that allow user to create a desired configuration using drag and drop modular environment. GSP demands ample technical understanding of the gas turbine from the user while GasTurb urges very less understanding of the gas turbine operators. However, there is a tradeoff between the benefits of both programs in different domains. 

In the beginning of the 21st century, Alexiou and Mathioudakis [216] from Laboratory of Thermal Turbomachines Greece developed another commercial software PROOSIS, that is quite modular in nature having a reusable library of GT components through a graphical user interface. Apart from this, auxiliary components of GTs such as gear boxes, generators, and propellers also exist in the component library that make the software very flexible and accurate in terms of transient modeling. Additionally, this software contains the inherent ability of developing a frequency response analysis due to variation of input fuel and demonstrating performance adaptation factors through matching the model with the available measurements [217]. Similarly, GTAnalysis was also built by Technological Institute of Aeronautics (ITA), Brazil. This program is also user friendly and modular in nature due to interactive block structuring that help in required modification [218]. One thing that differentiates GTAnalysis from other software is, it can accommodate variable geometry angle setting of the turbine as well as compressor section, i.e., NGV can be staggered at desired angle.

A comparison of some commercial software has been listed in the Table 6. Variety of commercial and limited use in-house built software have been employed in the literature for transient modeling and validation of the developed models, i.e., GateCycle [40,45], DESTUR [26], GETRAN [58,219], TURBOMATCH [51,220], AMESim [42], Thermoflow [7], DYNGEN [32,221]. TERTS [222], TURBOTRANS [223,224], and FORTRAN based programs [50,51,206,225].

### 6.2. Multi-Dimensional Simulation Programs

The simulations programs that considers physical dimensions such as radial and circumferential, along with information of gas conditions at each station of the engine are known as multi-dimensional gas turbine simulation programs. Normally, these simulation programs can model the entire engine systems by incorporating details of the geometry of each component and blade rows information of turbomachinery components. One such program, numerical propulsion system simulation (NPSS) [234], was developed by NASA Glenn Research center to carry out design and analysis of aircraft engines and space transportation components at reduced time and effort avoiding the need of higher expenses for experimental test rigs on jet engine [235]. In this way, NPSS served as a numerical test rig for engine design engineers and operators to simulate engine performance overnight in cost effective manner. This software holds the inherent capability of analyzing different engine components simultaneously; can perform structural and aerodynamic analysis concurrently and at faster time steps. The object-oriented nature of the code developed in C++ environment assists in easy and quick integration of the other subsidiary codes such as compressor, combustor or turbine’s individual codes and any newly built code that belong to any other language or environment. Apart from this, the ‘zooming’, feature was added to the NPSS to get an in depth and detailed insight into the performance of the each engine component closely [236]. Using this feature design engineers and operator can get a better component wise analysis inside the system rather than by isolating the components. Lately, Argote et al. [233] have extended the already build code by incorporating volume dynamics element that can be utilized as a cycle solution during transient modeling. This updated model was used for a simple turbojet engine with simple duct system in order to check the effect of added volume geometries on the time step and input. Hence, the volume dynamics results from the turbojet model were verified with academic studies of volume dynamics with simple duct architecture. However, still there is room for further research to extend the model for other configuration engines. 

## 7. Future Recommendations

Nowadays, the reliability of the gas turbines is of paramount importance due to global economic crisis. The urge for prognostics, fault detection and diagnostics, and predictive condition monitoring demands a high accuracy in the computational modeling codes of gas turbines. As a result, an accurate and robust transient modeling simulation code plays a crucial role in ensuring a reliable and safe engine operation by preventing unplanned shutdown. The purpose of this comprehensive review is typically based on accumulation of all the necessary information regarding the transient modeling of variable geometry industrial gas turbines. In addition to this, some hidden threads are also reviewed that are compulsory for carrying out an accurate transient modeling, i.e., (i) exploration of various VIGV and bleed schedules from the literature, (ii) formulation of framework for selection of suitable schedule for every configuration with respect to gas turbine utility purpose, and (iii) data compilation of shaft’s polar moment of inertia for various configuration engine. Hence this comprehensive review will serve as a supporting document for the future researchers and gas turbine’s operation and maintenance staff in accurate and timely prognostics, fault detection and diagnostic (FDD), and predictive condition monitoring of variable geometry IGTs.

## 8. Conclusions

The purpose of this paper is to compile and critically analyze the aspects in transient modeling and simulation of variable geometry industrial gas turbines. Critical analysis of the available literature is carried out with the hope of identifying potential topics for further research. In general, it is found that some models are pale in comparison to others. The overall transient regimes have been reviewed comprehensively along with all the transient modeling techniques adopted in the literature. In addition, VIGV and VBV schedules have compiled from the open literature in order to build a database and framework for selection of suitable schedules according to the particular configuration of engine under modeling for accurate and efficient results. Moreover, different commercially available software simulations tools have been critically analyzed. However, the specific conclusions that are considered highly beneficial are as follows:

- Although, variety of pertinent transient models exits in the literature, there is a scarcity in transient models for variable geometry IGT.
- Control mechanisms associated with VIGV and VBV are indispensable for different transient regimes, i.e., startup load change and shutdown.
- The VIGV schedule selection framework along with VIGV schedule database play a vital role in academic modeling and real time operation and maintenance of IGT. For instance, framework can be beneficial during maintenance for proper calibration of a drifted VIGV schedule.
- The polar moment of inertia for several configuration of IGTs delineates a paramount importance for accurate modeling of transient behavior in variable geometry IGT

## Figures and Tables

**Table 1:** Transient studies in literature with variable geometry consideration.

Author	Year	Variable Geometry Features		
		VIGVs or VSVs	Variable Bleed	NGVs or VGVs for PT
Mohammadian and Saidi, [56]	2019	✓	✓	
Montazeri-Gh et al. [57]	2018	✓		
Schobeiri, [58]	2018			✓
Mehrpanahi et al. [34]	2018	✓		
Wang et al. [59]	2018			✓
Silva et al. [60]	2018	✓		
Wang et al. [61]	2018			✓
Enalou, [62]	2017		✓	
Montazeri-Gh, [63]	2017	✓		
Wang et al. [43]	2017	✓		
Kim et al. [45]	2016	✓		
Kim et al. [64]	2015	✓	✓	
Barbosa et al. [65]	2012	✓		
Chacartegui et al. [66]	2011	✓		
Barbosa et al. [67]	2011	✓		
Panov et al. [68]	2009	✓		
Silva et al. [69]	2007	✓		
Sekhon et al. [36]	2006	✓		
Bringhenti et al. [70]	2006	✓	✓	✓
Camporeale et al. [71]	2006	✓		
Kim et al. [72]	2001	✓		
Kim et al. [73]	2001	✓		
Kim and Soudarev, [74]	2000		✓	
Blanco and Henricks, [75]	1998	✓		
Boumedmed, [76]	1997	✓		
Perretto, [77]	1997	✓		
Bettocchi et al. [33]	1996	✓		
Nava et al. [78]	1995			✓
Mehr-Homji and Bhargava, [39]	1992	✓		

**Table 3:** Critical review of different compressor modeling techniques in the literature.

Method	Researchers	Respective Equations	Benefits	Overall Drawbacks
Map Scaling Method	[68,149,151,152,153]	$PR=\frac{{\left(PR\right)}_{design}-1}{{\left(PR\right)}_{map,\;\;design}-1}\;\left[{\left(PR\right)}_{map}-1\right]+1$ $\dot{m}=\frac{{\dot{m}}_{design}}{{\dot{m}}_{map,\;\;design}}\times {\dot{m}}_{map}$ $\eta =\frac{\eta_{design}}{\eta_{map,\;\;design}}\times \eta_{map}$	1. Quite easy and simplest method to develop compressor map 2. Time saving	1. Not Applicable for variable geometry compressor 2. Limitation in selection of reference map 3. Not accurate for off-Design operation 4. This method overlooks the compressibility factor
Sequential Stage Stacking method	[137]	Flow coefficient, $\phi =\frac{C_{x}}{u}$ Pressure Coefficient, $\psi =C_{P}T_{t,in}\left(PR_{s}^{\frac{\gamma -1}{\gamma }}-1\right)/U^{2}$ Temperature Rise,$\;\xi =\frac{C_{p}\Delta T_{t}}{U^{2}}$ Stage Efficiency, $\eta =\frac{\psi }{\xi }$	1. Accurate performance prediction 2. Applicable for both fixed and variable geometry compressors	1.Problem in off design operations 2. problems during stalling and choking 3. Time consuming 4. It requires Gas path geometric data like stage mean radius and annulus area that are not provided by manufacturer
Modified Stage Stacking Method	[154]	${\dot{m}}_{i+1\;}={\dot{m}}_{i}$ ${\dot{m}}_{i+1}C_{x_{i+1}}+P_{i+1}A_{i+1}={\dot{m}}_{i}C_{x_{i}}+P_{i}A_{i}+F_{s}$ ${\dot{m}}_{i+1}H_{t_{i+1}}={\dot{m}}_{i}H_{t_{i}}+{\dot{W}}_{s}$	1. Flexibility in boundary conditions 2. Time saving calculations due to simultaneous solutions 3. Variable geometry treatment by variation in setting angle 4. Applicable for transient modeling due to stability in numerical methods	1.Gas path geometric data like stage mean radius and annulus area are not provided by Manufacturer 2. Unavailability of reference data at Max efficiency
	[12,72,73,90,155,156]	$\psi^{*}=\psi_{Max}^{*}-\frac{\psi_{Max}^{*}-1}{{\left(\phi_{\psi }^{*}{}_{Max}-1\right)}^{2}}\times {\left(\phi_{\psi }^{*}{}_{Max}-\phi^{*}\right)}^{2}$ $\eta_{Min}^{*}=1-\frac{1-\eta_{{\left(\frac{\psi }{\phi }\right)}_{Min}}^{*}}{{\left[1-{\left(\frac{\psi^{*}}{\phi^{*}}\right)}_{Min}\right]}^{3.5}}{\left(1-\frac{\psi^{*}}{\phi^{*}}\right)}^{3.5},\;where\;\frac{\psi^{*}}{\phi^{*}}\in \left[{\left(\frac{\psi^{*}}{\phi^{*}}\right)}_{Min},\;1\right]$ $\eta_{Max}^{*}=1-\frac{1-\eta_{{\left(\frac{\psi }{\phi }\right)}_{Max}}^{*}}{{\left[{\left(\frac{\psi^{*}}{\phi^{*}}\right)}_{Max}-1\right]}^{3.5}}{\left(\frac{\psi^{*}}{\phi^{*}}-1\right)}^{2},\;where\;\frac{\psi^{*}}{\phi^{*}}\in \left[1,\;{\left(\frac{\psi^{*}}{\phi^{*}}\right)}_{Max}\right]$	Thermodynamic cycle program combined with performance maps generated through stage stacking helps in searching the operating point values (thermodynamic performance parameters) like compressor PR, SFC and TET that is not possible in the rest of the methods	The Minimization Algorithm adopted in this method is not stable for large number of unknowns. So a robust technique i.e., Genetic Algorithm is suggested for future work
Blade Element Method	[75,157,158]	$\Delta h_{t}=(U_{2}^{2}-U_{2}C_{x2}\tan\beta_{2}-U_{1}C_{x1}\tan\alpha_{1})/g_{c}$ $\bar{\omega }=\frac{P_{t1}-P_{t2}}{P_{t1}-P_{1}}$ $C_{D}=\frac{\bar{\omega }}{\sigma }\left(\frac{\cos^{3}\alpha_{m}}{\cos^{2}\alpha_{1}}\right),Where\;\tan\alpha_{m}=\left(\frac{\tan\alpha_{1}+\tan\alpha_{2}}{2}\right)$	1. Little dependency on the cascade data 2. Applicable for variety of stages of compressors according to the desired compressor 3. Holds good for VIGV adjustment due to simulation of each blade element	The loss and deviation correlation curves obtained from the literature are not robust in terms of accuracy

**Table 4:** Turbine modeling equations.

Turbine Modeling Methods	Respective Mathematical Expression	Significance
Choking Equation	$\frac{{\dot{m}}_{in}\sqrt{T_{in}}}{\kappa AP_{in}}=Constant$ Where $\kappa =\sqrt{\frac{\gamma }{R}}{\left(\frac{2}{\gamma +1}\right)}^{\frac{\gamma +1}{\gamma -1}}$	An alternative of turbine performance maps. Gives low operational expansion ratio during start up process
Stodola Ellipse Equation	$\frac{m_{in}\sqrt{T_{Tin}}}{P_{in}}=K\;\sqrt{1-{\left(\frac{P_{out}}{P_{in}}\right)}^{2}}$	Useful for estimating turbine characteristics during off design condition
Flugel Formula	$\frac{m_{in}}{m_{in,ref}}=\frac{\sqrt{{\left(p_{in}-p_{out}\right)}^{2}}}{\sqrt{{\left(p_{inref}-p_{outref}\right)}^{2}}}\times \frac{\sqrt{T_{inref}}}{\sqrt{T_{in}}}\;$	Gives a correlation of mass flow, pressure and temperature for turbine in off design condition

**Table 5:** Different Correction factors for compressor map generation.

Researcher	Correlations	Correction Factors
Celis et al. [193]	Mass flow ${\left[\frac{\left(\frac{\dot{m}}{p_{in}}\times \frac{\sqrt{RT_{in}}}{\gamma }\right)}{{\left(\;\frac{\dot{m}}{p_{in}}\times \frac{\sqrt{RT_{in}}}{\gamma }\right)}_{DP}}\right]}_{VIGV}=a{\left[\frac{\left(\frac{\dot{m}}{p_{in}}\times \frac{\sqrt{RT_{in}}}{\gamma }\right)}{{\left(\;\frac{\dot{m}}{p_{in}}\times \frac{\sqrt{RT_{in}}}{\gamma }\right)}_{DP}}\right]}_{n}$ Pressure ratio ${\left[\frac{\frac{p_{out}}{p_{in}}-1}{{\left(\frac{p_{out}}{p_{in}}-1\right)}_{DP}}\right]}_{VIGV}=b\;{\left[\frac{\frac{p_{out}}{p_{in}}-1}{{\left(\frac{p_{out}}{p_{in}}-1\right)}_{DP}}\right]}_{n}$ Efficiency ${\left[\frac{\eta }{{\left(\eta \right)}_{DP}}\right]}_{VIGV}=c\;{\left[\frac{\eta }{{\left(\eta \right)}_{DP}}\right]}_{n}$	Correction factor a, b and c represents change in mass flow, pressure ratio and efficiency, respectively.
Kurzke, [194]	${\dot{m}}_{VIGV}={\dot{m}}_{FG,map}\times \left(1+\frac{c_{1}\Delta \theta_{VIGV}}{100}\right)\;$ ${\left(PR-1\right)}_{VIGV}={\left(PR-1\right)}_{FG,map}\times \left(1+\frac{c_{2}\Delta \theta_{VIGV}}{100}\right)$ $\eta_{VIGV}=\eta_{FG,map}\times \left(1-\frac{c_{3}\Delta \theta_{VIGV}^{2}}{100}\right)$	$c_{1}$,$\;c_{2}$,$\;c_{3}$
Knopf, [195]	Mass flow correction ${\dot{m}}_{OD}={\dot{m}}_{DP}\times \frac{P_{1,OD}}{P_{1,DP}}\times \frac{T_{1,DP}}{T_{1,OD}}\left(-K_{V}\Delta \theta_{VIGV}\right)\left[1+K_{T}\left(\frac{T_{1,OD}-T_{1,DP}}{T_{1,DP}}\right)\;\right]$ Efficiency correction $\eta_{OD}=\eta_{Max}\left(1-\left|\frac{{\dot{m}}_{DP}-{\dot{m}}_{OD}}{{\dot{m}}_{DP}}\right|\left(K_{m}\right)\right)\left[1+K_{E}\left|\frac{N_{OD}-N_{Max,\eta }}{N_{Max,\eta }}\right|\right]$	$K_{V}$,$\;K_{T}$,$\;K_{m}$,$\;K_{E}$
Plis and Rusinowski, [196]	Mass flow correction ${\dot{m}}_{VIGV}={\dot{m}}_{VIGV,Max}\left[1-K_{V}\left(\theta_{VIGV,Max}-\theta_{VIGV}\right)\right]$ Efficiency empirical correlation developed from Wirkowski’s [197] experimental data $\eta_{VIGV}=\alpha_{0}+\alpha_{1}\cdot {\dot{m}}_{corr}+\alpha_{2}\cdot {\dot{m}}_{corr}^{2}+\alpha_{3}\cdot N_{corr}+\alpha_{4}\cdot N_{corr}^{2}+\alpha_{5}\cdot {\dot{m}}_{corr}\cdot N_{corr}+\alpha_{6}\cdot \theta_{VIGV}+\alpha_{7}\cdot \theta_{VIGV}^{2}+\alpha_{8}\cdot {\dot{m}}_{corr}\cdot \theta_{VIGV}+\alpha_{9}\cdot N_{corr}\cdot \theta_{VIGV}$	$K_{V}$

**Table 6:** Comparison of various transient modeling commercial software tools.

Software	Developer/Owner	Type	Variable Geometry	Range of Flexibility	Pros	Cons	Reason for Utilization in Various Studies
GasTurb	Dr. Joachim Kurzke	0 D, OOP	VIGV + Bleed schedule	1. Turbomachinery fouling and erosion, 2. Inlet flow distortion, 3. Optimization, 4. Monte-Carlo	1. Need limited information from user, 2. User friendly for the GT operators due to predefined engine configuration	The model cannot be saved and transferred. For some cases it is very hard to import excel file, VIGV schedule only with respect to speed	Validation [43,50,145,226,227,228]
GSP	National Aerospace Laboratory NLR, Netherlands	0D, OOP	VIGV +Bleed schedule	Turbomachinery fouling and erosion, shaft dynamics,	1. Easy saving and transporting of the model to other PCs, 2. Effects of ambient and flight conditions, installation losses, and malfunctions of control can be simulated	It is not user friendly for the gas turbines operators because its need every detail from the user even configuration need to be structured by user	Validation [44,49,222]
PROOSIS	Alexiou from National Technical University of Athens	1D, OOP	No VIGV and Bleed	Parametric study, Optimization, Diagnostics	1. Model adaptation to specific engine using measured data 2. Frequency response analysis can be performed 3. Availability of extra auxiliary components such as gear box, generator and propeller	creation of libraries in the EL language require good expertise in the mathematical formulation of the components that limit this software to only academic community not to operators	validation [9,23,45,229]
GTAnalysis	Gas Turbine Group, ITA, Brazil	Modular with interactive block structuring	VIGV and Bleed schedule, VAN for turbines	Deterioration	Due to its modular characteristic, any required modification can be easily incorporated to the program, making it very friendly	For variable geometry effect study, another in house program AFCC need to utilize	Modeling [14,60,69,70,230]
NPSS	NASA Glenn Research Center	multi-D	No	1. Integration of components for large systems and subsystems 2. effects of aerothermal and structural loadings on geometry and efficiency can be simulated	1. Additional codes can be appended 2. Zooming can give more details of the component performance inside the engine 3. High fidelity variable complexity analysis can be performed during design problems	Only available to partner research institutes of NASA	Modeling [184,231,232,233]

## Nomenclature

Nomenclature		Max	Maximum
e	Internal energy per unit mass	Min	Minimum
F	Force	M	Mean value
F	Force Vector	to	total
H	Total enthalpy	to,in	total inlet
$\dot{m}$	mass flow rate	cc	combustion chamber
n	unit vector normal to the surface	DP	Design Point
p	static pressure	VIGV	VIGV corrected value
$\dot{Q}$	Heat transfer	FG,map	Fixed Geometry compressor map
S	Surface	$a,b,c,c_{1},c_{2},c_{3},$ $K_{v},K_{T},K_{m},K_{E}$	VIGV correction factors
t	time	OD	Off design
A	Area	Max,η	Maximum efficiency value
u	Axial velocity	corr	corrected value
u	velocity vector	corr,map	corrected values from maps
V	Volume	dis	discharge value
$\dot{W}$	power	v,max	maximum extent of bleed valve opening
ω	angular velocity	Greek Letters	
J	Polar moment of inertia	ρ	density
N	shaft rotational speed	$\tau_{I}$	Inertial time constant
m	mass	Δ	Change
r	Engine radius	$\tau_{c}$	Compressor Torque
P	Total Pressure	η	isentropic efficiency
T	Total temperature	γ	polytropic index
R	Gas Constant	ϕ	flow coefficient
$C_{p}$	specific heat at constant pressure	ψ	pressure coefficient
d	diameters	ξ	temperature rise coefficient
U	tangential blade speed	α	flow angle relative to a stator
$PR_{s}$	stage pressure ratio	β	flow angle relative to a rotor
G	Generalized functions	$\tau_{cc}$	burner time constant
a	numbers of variables at each control surface	$\theta_{VIGV}$	VIGV angle
Z=3(n+1)	total number of variables	$\theta_{VAN}$	VAN angle
$C_{D}$	Drag Coefficient	$\bar{\omega }$	Total pressure loss coefficient
h	enthalpy	σ	Pressure ration between compressor inlet and sea level
K	Stodola Constant	Abbreviations	
κ	Choking constant	SF	shape factor
u(t)	input signal	CCPP	combined cycle power plant
y(t)	Output signal	FDD	fault detection and diagnostics
r(t)	Demanded signal	GA	Genetic algorithm
e(t)	Error signal	GG	Gas Generator
$K_{p}$	proportional controller gain	HRSG	Heat Recovery steam generator
$K_{i}$	integral controller gain	ICV	Inter-component volume
$K_{d}$	derivative controller gain	CMF	Constant Mass Flow
$C_{g}$	extent of bleed valve opening	IGT	Industrial gas turbines
Subscripts		VIGV	Variable inlet guide vane
i	control volume index	VSV	Variable Stator vane
in	inlet	VAN	Variable area nozzle
out	outlet	VBV	variable bleed valve
t	turbine	NGV	Nozzle guide vane
c	compressor	ODEs	Ordinary Differential equations
f	fictional power loss	PDEs	partial differential equations
el	electric load	OOP	Object oriented programming
s	shaft	TIT	Turbine inlet temperature
d	design	TET	Turbine exhaust temperature
target,Eng	Target Engine	HPC	High pressure compressor
ref,Eng	Reference Engine	HPT	High pressure turbine
GGS	gas generator shaft	LPT	Low pressure turbine
PTS	Power turbine shaft	ANN	Artificial neural network
1	transition stage between to control volume	CFD	Computational fluid dynamics
a	air	CMs	continuity models
g	Gas	TFMs	Transfer function models
in,d	intake design point value	LFMs	Linear function models
1,2,3,4	station numbers		
